# Into the dynamics of rotaxanes at atomistic resolution†

**DOI:** 10.1039/d3sc01593a

**Published:** 2023-05-30

**Authors:** Luigi Leanza, Claudio Perego, Luca Pesce, Matteo Salvalaglio, Max von Delius, Giovanni M. Pavan

**Affiliations:** a Department of Applied Science and Technology, Politecnico di Torino Corso Duca degli Abruzzi, 24 10129 Torino Italy giovanni.pavan@polito.it; b Department of Innovative Technologies, University of Applied Sciences and Arts of Southern Switzerland, Polo Universitario Lugano Campus Est, Via la Santa 1 6962 Lugano-Viganello Switzerland; c Department of Chemical Engineering, University College London London WC1E 7JE UK; d Institute of Organic Chemistry, Ulm University Albert-Einstein-Allee 11 89081 Ulm Germany

## Abstract

Mechanically-interlocked molecules (MIMs) are at the basis of artificial molecular machines and are attracting increasing interest for various applications, from catalysis to drug delivery and nanoelectronics. MIMs are composed of mechanically-interconnected molecular sub-parts that can move with respect to each other, imparting these systems innately dynamical behaviors and interesting stimuli-responsive properties. The rational design of MIMs with desired functionalities requires studying their dynamics at sub-molecular resolution and on relevant timescales, which is challenging experimentally and computationally. Here, we combine molecular dynamics and metadynamics simulations to reconstruct the thermodynamics and kinetics of different types of MIMs at atomistic resolution under different conditions. As representative case studies, we use rotaxanes and molecular shuttles substantially differing in structure, architecture, and dynamical behavior. Our computational approach provides results in agreement with the available experimental evidence and a direct demonstration of the critical effect of the solvent on the dynamics of the MIMs. At the same time, our simulations unveil key factors controlling the dynamics of these systems, providing submolecular-level insights into the mechanisms and kinetics of shuttling. Reconstruction of the free-energy profiles from the simulations reveals details of the conformations of macrocycles on the binding site that are difficult to access *via* routine experiments and precious for understanding the MIMs' behavior, while their decomposition in enthalpic and entropic contributions unveils the mechanisms and key transitions ruling the intermolecular movements between metastable states within them. The computational framework presented herein is flexible and can be used, in principle, to study a variety of mechanically-interlocked systems.

## Abstract 2

Free-energy profiles, and associated enthalpic and entropic components, providing atomistic-resolution insights into the mechanisms (thermodynamics and kinetics) of the shuttling processes in rotaxanes.

## Graphical abstract

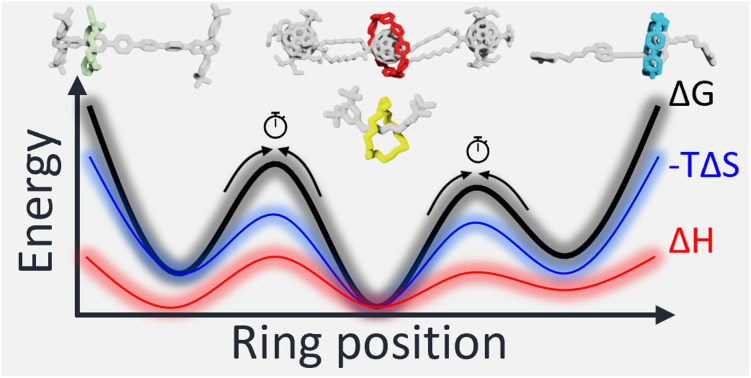

## 1 Introduction

Motor proteins are ubiquitous in nature and are at the basis of many biological processes, such as, *e.g.*, DNA replication,^1^ muscle contraction^2–5^ and ATP synthesis.^6–8^ These are complex molecular systems composed of molecular sub-parts that interact *via* non-covalent interactions, and move with respect to each other generating motion upon absorption of energy or external stimuli.^9^ For the last decades, an important challenge for chemists and physicists has been to design and synthesize artificial molecular machines (AMMs) with controllable movements, mimicking nature's technology.^10–12^ Great efforts in this field have produced a large variety of, *e.g.*, mechanically interlocked molecules (MIMs),^13–16^ such as, to mention a few, catenanes,^17^ rotaxanes,^18^ and molecular shuttles,^15^ and have led to the Nobel Prize for Chemistry awarded to Feringa, Sauvage and Stoddart in 2016.

Catenanes consist of two or more entangled rings forming a mechanically interlocked chain, while rotaxanes and shuttles typically consist of one or more macrocyclic rings mechanically interlocked around a dumbbell-shaped molecule (the axle) with two bulky groups at both ends (stoppers). In rotaxanes, the macrocycles can move back and forth along the axle, and we take the liberty to describe this translational movement as “shuttling”. The same type of linear movement occurs also in the so-called “molecular shuttles”, while the fundamental difference of a shuttle (as opposed to a simple rotaxane) is that there are at least two distinct binding sites for the ring within the thread, such that there is not one single global thermodynamic minimum for the stochastic location of the ring (but at least two local minima). As a relevant example, Green *et al.*^19^ designed a monolayer of bistable molecular switches as a storage element, relating the relaxation kinetics with the memory retention time of the device. Significant breakthroughs were also achieved, for example, in catalysis, exploiting the threading of the macrocycle along the thread to expose/hide organocatalytic groups,^20–23^ or in drug delivery, where the controlled disassembly of a biocompatible [2]-rotaxane was exploited to trigger the release of anticancer drugs.^24^

Designing MIMs with controllable dynamical properties could benefit from a deeper understanding of the factors controlling their structure and dynamics. In particular, reaching a molecular understanding of rotaxane dynamics in solution and unraveling the details of the mechanisms (thermodynamics and kinetics) underpinning their behavior is crucial for designing MIMs with desired properties. Experimental investigations based, for example, on nuclear magnetic resonance, coalescence methods, or cyclic voltammetry allowed estimating the dynamics of rotaxanes and molecular shuttling. For example, the influence of the axle length,^25,26^ the conformational flexibility of spacers^27,28^ and of different environments^29,30^ on the shuttling rate constants were extensively investigated. However, the spatio-temporal resolution of such kinetic experiments is typically limited, making it difficult to obtain direct information on the molecular factors that control the dynamics of these systems. To this end, it would be necessary to study the dynamics of the MIMs at a sub-molecular resolution.

Molecular models and computer simulations are useful to this end, as they may provide a direct and detailed viewpoint on the atomistic/molecular mechanisms that regulate the dynamics of MIMs.^31–43^ Molecular Dynamics (MD) simulations have been used, for example, to investigate the effect of the solvent and the surrounding environment modulating the thermodynamics of various rotaxanes.^44–50^ However, in many MIMs the shuttling occurs on timescales that largely exceed those accessible *via* classical atomistic MD simulations. Enhanced sampling techniques (such as, *e.g.*, metadynamics,^51^ adaptive bias force,^52^*etc.*) are useful in such cases, allowing to investigate rare transition events that occur on experimentally-relevant timescales at atomistic/sub-molecular resolution.^53–61^ For example, metadynamics (MetaD) simulations have been recently used to investigate the dynamics of monomer exchange in supramolecular polymers^58–60^ or the innate guest exchange dynamics in-and-out the cavity of a coordination cage,^61,62^ as well as the motion and dynamic behaviors of supramolecular systems (*e.g.*, nanoparticles, tubules) in out-of-equilibrium conditions or under the effect of an external stimulus.^63,64^ Offering the opportunity to study molecular motion events occurring on long timescales at atomistic resolution and providing precious insights on the key steps or factors controlling them, these approaches are well suited also to study the dynamics of MIMs such as, *e.g.*, rotaxanes and molecular shuttles.

Here, we combine standard MD and well-tempered metadynamics (WT-MetaD) simulations^65^ to investigate at the atomistic level the thermodynamic and kinetic behavior of four [2]rotaxanes,^25,28,66,67^ used as representative examples and covering different features and architectures in the framework of MIMs. With our *in silico* approach, we obtain a detailed insight into the thermodynamics of these systems, obtaining results that are consistent with the experimental evidence. We reconstruct the thermodynamics and kinetics of these innately dynamic systems. A decomposition of the free-energy profiles into the enthalpic and entropic components^68,69^ provides detailed insights and an atomistic comprehension of the key mechanisms controlling the intrinsic dynamic behavior of such systems. The computational framework described herein is general and may open the way toward obtaining direct structure-dynamics relationships for a variety of mechanically interlocked molecular systems.

## 2 Results and discussion

Herein, we used different types of [2]rotaxanes as representative case studies in the effort of obtaining a general-purpose approach capable of exploring the key factors controlling the variegated dynamics of MIMs.

### 2.1 Mechanism and dynamics of shuttling of a [R_4_ − H_2_]^2+^ [2]rotaxane

**Fig. 1:**
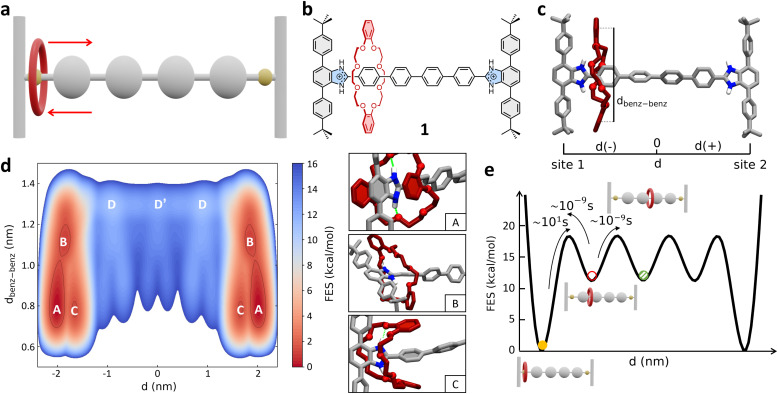
Mechanism of shuttling in the [R_4_ − H_2_]^2+^ [2]rotaxane (1). (a) Schematic representation of the mechanism of shuttling and (b) structural, chemical formula of the dicationic [2]rotaxane 1. (c) Atomistic molecular model of 1. Two collective variables (CVs) are used in the WT-MetaD simulations – CV1: *d* (distance between the geometrical center of the DB24C8 ring and the center of the rotaxane axle), and CV2: *d*_benz–benz_ (distance between the centers of the two benzene rings of the shuttling macrocycle). Only the hydrogen atoms of the recognition sites are displayed, while the other ones in the rotaxane are not shown for clarity. (d) Left: FES of the shuttling process obtained from WT-MetaD and represented as a function of CV1 (*x*-axis: symmetric respect to the axle center – see also Methods section in the ESI† for further details) and CV2 (*y*-axis). Right: Insets showing three representative snapshots of states A, B, and C (relevant states in the FES on the left). (e) Scheme of the FES diagram (as a function of *d*) detailing the Δ*G* differences between the various conformational states – obtained *via* well-converged WT-MetaD recrossing between the various states – the transition barriers Δ*G*^‡^, and the related characteristic transition timescales *τ* – estimated from multiple infrequent WT-MetaD simulations (see also Methods section in the ESI†). The free-energy of each state (colored circles) as well as the height of free-energy barriers between them are defined with respect to state A (global minima, set to 0).

As a first case study, we focus here on the H-shaped dicationic [R_4_ − H_2_]^2+^ [2]rotaxane (referred to hereinafter as 1, Fig. 1b) reported by Gholami *et al.*^25^ System 1 consists of a rigid dumbbell with four phenyl rings between two benzimidazolium recognition sites, and a dibenzo[24]crown-8 ether (DB24C8) macrocycle, which instead is a relatively flexible element. This system has been previously taken as a reference for studying the influence of the axle length on the rate of shuttling in neutral and dicationic molecular shuttles. Furthermore, kinetic shuttling experiments have been conducted providing rate measurements that can be compared to computational results.^25^

As a first step, we built an all-atom (AA) model for 1 (Fig. 1a–c). This was parametrized according to the General Amber Force Field (GAFF) – complete details on the AA models used herein and on their parametrization are provided in the Methods section in the ESI.† The 1 AA model was then immersed in a periodic simulation box filled with explicit acetonitrile (ACN) solvent molecules (the same solvent used in the experiments of ref. 25) and neutralizing PF_6_^−^ counterions. The system was then equilibrated *via* a preliminary MD simulation (details on the simulations conducted herein are provided in the Methods section in the ESI†).

Since at room temperature (298 K) the shuttling of this system occurs on longer timescales than those efficiently sampled *via* classical MD simulation, we turned to Well-Tempered Metadynamics (WT-MetaD) simulations.^65^ In WT-MetaD, standard MD is modified by introducing a history-dependent bias potential properly designed to drive the system in the exploration of the most relevant equilibrium conformations (and transitions) along one or more critical reaction coordinates represented by collective variables (CVs). In our case we are primarily interested in using WT-MetaD simulations to study the shuttling within rotaxane 1. Our tests demonstrated that two CVs – *i.e.*, the location *d* of the geometrical center of the DB24C8 ring along the rotaxane axle (CV1), and the distance *d*_benz–benz_ between the centers of the two benzene rings of the shuttling macrocycle (see Fig. 1c and the scheme in Fig. S1†) – can appropriately describe and sample the shuttling mechanism in the rotaxane, allowing recrossing between all metastable states and robust reconstruction of the free-energy profile (see ESI Movie 1†).

Fig. 1d shows the free-energy surface (FES) of the shuttling process represented as a function of the two collective variables CV1 (*d*) and CV2 (*d*_benz–benz_). The WT-MetaD simulation unequivocally reveals that the most stable conformations of the rotaxane correspond to states where the DB24C8 macrocycle is located close to the lateral recognition sites (states A, B and C: |*d*| ≳ 1.5 nm), while the shuttling is characterized by significantly less favored intermediate states (Fig. 1d: states D, D′). A deeper inspection of the structure of these minima shows that they are quite broad along the *d*_benz–benz_ coordinate. This indicates that these two broad mirror minima at *d*_benz–benz_ ∼ 0.8 nm identify both states in which the macrocycle folds, stacking its benzene rings with the phenyl rings of the stoppers (state A, in Fig. 1d) or with the first central phenyl ring of the axle (state C, in Fig. 1d), and states in which the macrocycle is extended but still bound to the recognition site (state B, in Fig. 1d).

**Fig. 2:**
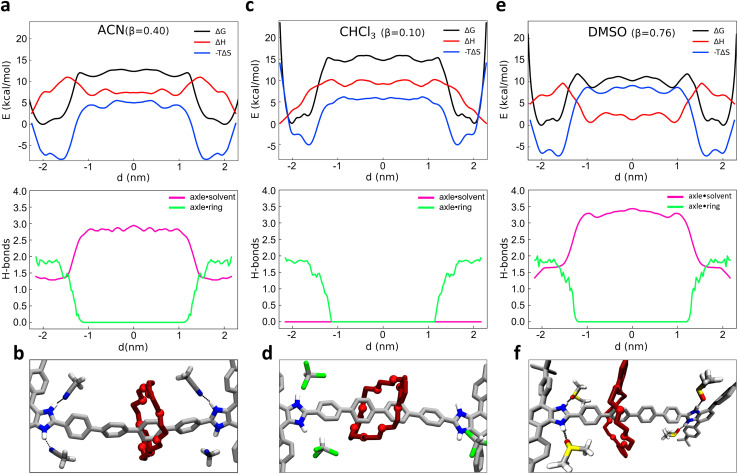
Role of solvent in the shuttling mechanism of 1. (a) Top panel: free-energy (black), enthalpy (red) and entropy (blue) profiles of the rotaxane 1 immersed in ACN solvent (*β* = 0.40) as a function of *d*; bottom panel: number of HBs between the axle and the macrocycle (green) and between the axle and the solvent molecules (purple line), as a function of *d*. (c and e) Same as (a), for CHCl_3_ (*β* = 0) and DMSO solvent (*β* = 0.76). (b, d and f) Representative view explaining the role of solvent in the shuttling of macrocycle: when the macrocycle (in red) is in “unbound” state, the solvent molecules surround the binding site: ACN (panel b) and DMSO (panel f) replace the ring in the formation of HBs with the benzimidazolium site (black dashed circles); conversely, this is not the case of CHCl_3_ molecules (panel d).

The A, B and C states correspond to “bound” conformations of the rotaxane, characterized by the presence of NH⋯O hydrogen bonds (H-bonds) between the macrocycle and the benzimidazolium groups present in the recognition sites (Fig. 1d, right: insets). The shuttling process thus requires that the DB24C8 ring breaks such HBs reaching an “unbound state” D from which it can then diffuse along the axle (see also further discussion below). As shown in Fig. 2a (bottom panel), the number of H-bonds drops from ∼2 for |*d*| ≳ 1.5 to 0 for 0 ≲ |*d*| ≲ 1.5 nm, while after breaking the interaction with the stoppers and leaving the bound states the DB24C8 ring diffuses along the axle (see also Fig. S2a:† plot of *d*_benz–benz_*vs. d*). Interestingly, once in the unbound state (Fig. 1d: D and D′), the macrocycle does not maintain an open conformation and the ring shuttling is not resistance-free due to steric effects related to the *para*-phenylene structure of the axle, to possible π–π interactions with the rings of the DB24C8 macrocycle, and to residual solvophobic effects. The plot of the number of contacts between the *ortho*-phenylene units of DB24C8 and *para*-phenylene units of the axle of Fig. S2b† indicates there are interactions between DB24C8 and the axle. During the shuttling, the DB24C8 macrocycle distorts assuming a clip-mode conformation in such a way as to promote π–π interactions between its rings with those of the axle (Fig. S2:† points where *d*_benz–benz_ decreases the number of DB24C8 × axle contacts increases). This result, which is also consistent with DFT calculations,^25^ demonstrates a step-wise diffusion of the macrocycle along the axle.

A well-converged WT-MetaD simulation, allowed us to reconstruct the FES associated with the system. This provided us with a reliable estimation of the free-energy differences (Δ*G*) between all the “bound” and “unbound” states that the system visits multiple times during the simulation. However, due to its high deposition rate, the energy bias can be deposited over the transition states and can affect the kinetic barrier. Additionally, the Δ*G* profile obtained from the simulation (Fig. 2a, top panel: Δ*G*, black curve) shows low barriers between intermediate states, suggesting that the set of variables is well suited to describe the translational motion of the macrocycle, but may not be optimal for describing the transition from the bound state. Therefore, we used a different strategy to estimate the transition barriers (Δ*G*^‡^) and kinetic information on the shuttling dynamics (*τ*: characteristic transition timescales between the various states).^53,56^ As recently done for studying rare transitions in other supramolecular systems,^58,60,63^ we ran multiple infrequent WT-MetaD simulations where the transition between metastable states is accelerated/biased and can thus be efficiently sampled. Provided that the simulations' setup is appropriate, and the CVs are able to distinguish among different basins, it is then possible to reconstruct the unbiased/native transition kinetics from the infrequent WT-MetaD biased one.^53,56,58^ In particular, we ran multiple infrequent WT-MetaD simulations activating the transition from main “bound” minimum A (Fig. 1d) to the “unbound” state (D).

This analysis provided a characteristic timescale for such a “rare” bound-to-unbound transition in the order of ∼tens of seconds (see Fig. S3a†), and an associated transition barrier of ∼18.5 ± 0.4 kcal mol^−1^. Repeating such analysis starting from bound states B or C state and activating the transition to D does not change the results, providing very similar kinetics and, globally, transition timescales in the same order of magnitude (see ESI, Fig. S3a†). The transitions between the “bound” states (A, B and C) are in fact much faster than that of the bound-to-unbound transition (ESI Movie 1†). In fact, the latter requires the rupture of the HBs with benzimidazolium and is thus the rate-limiting step for the shuttling from one station to the other (for this reason, from this point on we refer simply to the “bound” to “unbound” transition, as the distinction between A, B and C states is negligible for the shuttling dynamics).

The macrocycle shuttling along the axle (D → D′, D′ → D, *etc.* transitions) requires crossing lower barriers (Δ*G*^‡^ ∼ 6.0 ± 0.3 kcal mol^−1^) and are in comparison much faster (Fig. 1e). In particular, such intermediate shuttling transitions are found to occur in the timescale of nanoseconds (see also Fig. S3b†) and, contrary to the bound-to-unbound transition that requires a biased approach to be sampled, can be also efficiently observed *via* classical MD simulations in this system. These results are consistent with the experimental evidence. Experimental ^1^H NMR investigations revealed that the shuttling dynamics in 1 (passage of the macrocycle from one binding site to the opposite one) is characterized by a free-energy barrier in the order of ∼19.8 kcal mol^−1^,^25^ confirming the reliability of the modelling results and the appropriateness of the adopted simulation setup.

The results also demonstrate that the shuttling in such systems is essentially controlled by the characteristic rate for macrocycle unbinding, while the diffusion on the axle is in comparison extremely fast in these conditions (Fig. 1e: ∼9 orders of magnitude faster).

### 2.2 Enthalpic and entropic effects, and role of solvent on the shuttling of [R_4_ − H_2_]^2+^ [2]rotaxane

As recently shown *via* computational approaches,^43–50^ the shuttling dynamics and mechanisms in such kinds of rotaxanes may be significantly determined by the influence of the environment (*i.e.*, the solvent composition). It is thus interesting to characterize and compare the shuttling free-energy profiles for the system in different solvents. As test cases, we compared the dynamical behavior of system 1 as immersed in four different solvents. Given that the behavior of 1 is strongly controlled by the H-bonds between the macrocycle and the binding sites in the rotaxane, we chose to compare solvents having different *β* parameters (the hydrogen-bond acceptor ability):^70^ chloroform (CHCl_3_, *β* = 0.10), dichloromethane (DCM, *β* = 0.10), acetonitrile (ACN, *β* = 0.40) and dimethyl sulfoxide (DMSO, *β* = 0.76). While ACN and in particular DMSO solvents may thus have considerable local effects on the dynamics of the shuttling, by forming H-bonds with the axle and thus competing with those of the macrocycle, DCM and CHCl_3_ are “inert” in this sense, and may in principle contribute to the shuttling dynamics only *via* residual solvophobic effects. To explore in more detail the solvent impact and attain additional insight into the factors that regulate the equilibrium of the rotaxane, we also enriched our analysis through a breakdown of the free-energy profiles (Δ*G*) into enthalpic (Δ*H*) and entropic (−*T*Δ*S*) contributions. To this end, we employed a validated approach^68^ recently proven useful for the study of the early nucleation stages in metal–organic frameworks^69^ for obtaining a deeper insight into the behavior of such rotaxane in different *β* solvents.

Starting from the ACN case (acetonitrile has *β* = 0.40), a projection of the FES on the *d* variable clearly shows two main minima close to the recognition sites, at |*d*| ∼ 1.5–2 nm from the axle center (Fig. 2a, top: Δ*G*, black line). The free-energy difference Δ*G* between the bound minima and the unbound state in this system is found ∼11–12 kcal mol^−1^. We decomposed the FES (global free-energy, Δ*G*, in black) into the enthalpic and entropic terms, obtaining respectively the red (Δ*H*) and blue (−*T*Δ*S*) curves of Fig. 2a (top: the sum of red and blue curves gives the free-energy, in black). The Δ*H* profile (red) shows two minima in correspondence of the termini of the rotaxane. In those positions the macrocycle interacts with the rest of the rotaxane both *via* H-bonding with the benzimidazolium sites and *via* π–π stacking with the stoppers (as shown in Fig. 1d: topmost inset on the right), thus maximizing the solute–solute interactions in the system. The green curve of Fig. 2a (bottom) shows how upon macrocycle unbinding from the stoppers and diffusion along the axle the number of macrocycle–rotaxane HBs drops from ∼2 to 0 for |*d*| ≲ 1.5 nm. In particular, when the macrocycle leaves the station the system has to cross an enthalpy barrier to shuttling of ∼9 kcal mol^−1^.

The fact that the number of HBs between the macrocycle and the rest of the rotaxane remains ∼2 for |*d*| ≳ 1.5 nm indicates that such an enthalpy barrier is due on a first instance to the breakage of the π–π interactions between the macrocycle and the stoppers (unclipping) and on a second instance to the breakage of the macrocycle–axle HBs. It is worth noting that ACN molecules themselves can form HBs with the benzimidazolium sites of the rotaxane, taking an active (and competitive) role in the behavior of the rotaxane. The pink curve of Fig. 2a (bottom) shows that when the macrocycle is in one of the two stations (HBs ∼ 2 in green), the free benzimidazolium site is engaged in HBs with the ACN molecules (HBs ∼ 1.5 in pink), for a total of ∼3.5 HBs involving in the rotaxane in the bound state. This suggests that the HBs of the axle with the macrocycle are to some extent more stable than those with the ACN solvent molecules (∼2 *vs.* ∼1.5). Once the ∼2 HBs between macrocycle and the axle are broken (green HBs drop to 0) these are replaced by others ∼1.5 with ACN molecules during the shuttling (pink HBs rising ∼3 for states |*d*| ≲ 1.5). Fig. 2b shows a snapshot of the ACN–rotaxane HB interactions during macrocycle shuttling. This means that while the HBs with the ACN solvent compensate for the enthalpy penalty provided by the breakage of the HBs with the macrocycle, a net difference of ∼3.5 *vs.* ∼3 HBs is observed between the two states. Summed to the loss of the π–π interactions between the macrocycle and the stoppers during the macrocycle shuttling, this fits well with a Δ*H* of ∼5 kcal mol^−1^ between the bound *vs.* unbound macrocycle states observed in the red profile of Fig. 2a (top).

At the same time, with the rupture of the π–π interactions and of the HBs, the binding between the axle and macrocycle becomes less constrained, and the entropic term becomes more favorable (minima of the blue line in Fig. 2a, top). The blue profile of the entropic term is found rather similar to that of the FES (black), indicating that the behavior of such rotaxane is largely controlled by entropy in such conditions.

Similarly to what we have seen in ACN solvent, the free-energy profile computed in CHCl_3_ solvent (*β* = 0.10, Fig. 2c, top: black curve) also shows two minima close to the recognition sites (at |*d*| ∼ 1.5–2 nm from the axle center). Also in this case the Δ*H* profile (Fig. 2c, top: red) exhibits a global minimum in the configuration with the macrocycle clamped around the benzimidazolium terminal groups (*d* ∼ ±2.1 nm). However, in CHCl_3_ the Δ*H* difference between the macrocycle bound *vs.* unbound/shuttling states is found higher than in ACN solvent (∼ 10 kcal mol^−1^). In Fig. 2c (bottom) it is interesting to note that when the green HBs drop to 0 due to macrocycle unbinding from the terminal stations, in this case, no HBs involving the CHCl_3_ solvent molecules are possible, and the pink HBs curve remains equal to 0 everywhere (see also snapshot in Fig. 2d). In terms of the whole system (solute plus solvent), this makes the shuttling states less favorable than in ACN solvent in this case and consequently, the bound ones are more stable in CHCl_3_ than in ACN. Estimation of the unbinding free-energy barrier *via* infrequent MetaD calculations provided a Δ*G*^‡^ ≃ 21.2 ± 0.4 kcal mol^−1^ (see ESI, Fig. S4 and S6†) compatible with a slow shuttling rate (with an unbinding transition time in the order of minutes in CHCl_3_ solvent).

As additional tests, very similar results to those in CHCl_3_ are obtained when studying the system in DCM, which has *β* = 0.10 (as CHCl_3_ – see Fig. S4–S6†). On the contrary, the free-energy profile obtained for the same rotaxane in DMSO solvent (*β* = 0.76, even higher than that of ACN) suggests a behavior closer to that in ACN, and even more pronounced. As it can be observed from Fig. 2e (top panel), while showing a similar FES shape (*e.g.*, position of the black curve minima), the Δ*G* between the unbound and bound states is reduced to (∼9.5 kcal mol^−1^) in this case (and the free-energy barrier computed *via* infrequent WT-MetaD is reduced to Δ*G*^‡^ ∼ 16.5 ± 0.3 kcal mol^−1^: see ESI and Fig. S4 and S6†). These results fit with a faster shuttling kinetics of the macrocycle between the unbound and bound states. It is interesting to note how such a shuttling acceleration is related to a higher tendency of DMSO solvent molecules to accept HBs from the benzimidazolium site, thereby competing with the macrocycle and favoring the unbinding of the latter from the recognition site and the macrocycle shuttling. As shown in the bottom panel of Fig. 2e, the average number of HBs between the DMSO solvent molecules and the binding stations increases from ∼1.5 to ∼3.5, once the macrocycle starts shuttling. The formation of HBs between the recognition sites and DMSO molecules not only compensates for the energy loss caused by the rupture of HBs between axle and macrocycle but also determines an overall favorable enthalpic configuration: this is demonstrated by the global enthalpy Δ*H* minimum, which corresponds to states where the macrocycle is shuttling instead of bound at the stations (opposite to what happens, *e.g.*, in ACN or CHCl_3_) (Fig. 2e, top panel, red curve).

In terms of *β* value, ACN solvent (*β* = 0.40) is an intermediate case between the CHCl_3_/DCM and DMSO solvents. The Δ*G* between bound and unbound states of ∼12 kcal mol^−1^ and the free-energy barrier in ACN estimated *via* infrequent WT-MetaD of Δ*G*^‡^ ∼ 18.4 kcal mol^−1^ (see Fig. S6† and previous paragraph) are both in between high and low *β* solvents. Also, the average number of HBs between solvent and benzimidazolium (Fig. 2a, bottom panel) increases up to ∼2.9 units as the macrocycle begins the shuttling (*vs.* 0 units in CHCl_3_/DCM and ∼3.5 in DMSO). These evidences confirm that the dynamics of this system follows a clear trend dictated by the solvent propensity to form HBs. In particular, the stronger the propensity of the solvent molecules to establish HBs, the more pronounced is the solute–solute *vs.* solute–solvent HBs competition in the system.

These results show how such a simulation approach is useful to obtain a deep molecular-level insight into the shuttling mechanism, revealing how the external conditions (*e.g.*, the solvent used) may determine the process. To generalize the knowledge that can be obtained, we tested our approach on other types of MIMs.

### 2.3 Shuttling mechanism in a formamidinium [2]rotaxane

**Fig. 3:**
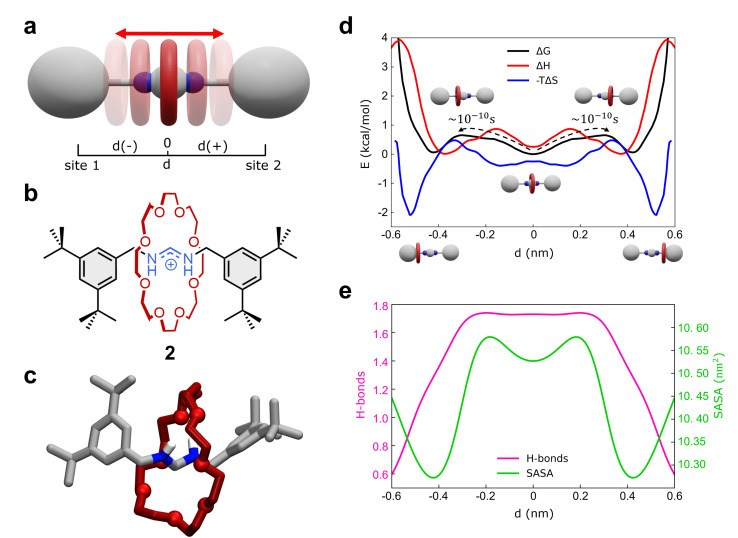
Shuttling dynamics of the formamidinium [2]rotaxane (2). (a) Schematic representation of the mechanism of shuttling along the translational position *d* of the macrocycle, and (b) structural, chemical formula of the formamidinium [2]rotaxane (2). (c) AA molecular model of 2. Only the hydrogens of the recognition sites are displayed for clarity. (d) Free-energy (black), enthalpy (red) and entropy (blue) profiles associated with the system. The associated transition times are estimated *via* single-transition MD simulations. (e) Number of HBs between the crown and the thread (purple line) and SASA of the rotaxane (green line) as a function of *d*.

As a second representative test case, we studied the formamidinium [2]rotaxane recently reported by Borodin *et al.* (see Fig. 3a–c).^66^ This rotaxane consists of an *N*,*N*′-disubstituted formamidinium ion and the flexible 24-crown-8 (24C8) ether (Fig. 3b), and it is part of a seminal work describing the self-assembly of stimuli-responsive [2]rotaxanes, using the dynamic covalent reaction amidinium exchange.^71^ This system, referred to hereinafter as 2, allows us to test our methods on a relevant case in which both the axle and the macrocycle are flexible.

In solution, system 2 exists as a mixture of *E*,*E* and *E*,*Z* isomers, which undergo fast isomerization. We built an AA model for the *E*,*E* isomer of rotaxane 2 (Fig. 3b and c), while isomerization may occasionally occur within the timescale accessible by simulations. This was then immersed in a simulation box filled with explicit DCM solvent molecules, the same solvent used in the experiments,^66^ and with weakly coordinating PF_6_^−^ anions (see Methods in the ESI† for additional details). After preliminary energy minimization and short equilibration MD conducted in NPT periodic boundary conditions, we studied the system through classical MD simulation. Enhanced sampling (MetaD) was unnecessary in this system, as the timescale of shuttling dynamics is very fast and accessible within a practically attainable MD timescale.

We first studied the translation of the macrocycle along the main axis of the axle molecule. The translational dynamics can be well described and monitored *via* the macrocycle position along the axle: *i.e.*, the coordinate *d* in Fig. 3a (see also ESI, Fig. S7a† for the definition of *d*). In this case, we could extract the FES directly from the unbiased MD trajectories, converting the histogram of the probability density. Fig. 3d (black line) reports the FES for the translational dynamics in this system as a function of *d*.

The FES shows three stable states along the coordinate *d*. The global minimum corresponds to the most stable conformation, with the macrocycle at the center of the axle (*d* ∼ 0 nm). This “central” state is flanked by two nearly identical lateral minima, just slightly higher than the central one in free-energy (∼+0.15 kcal mol^−1^). These lateral minima correspond to configurations where the macrocycle approaches the terminal stoppers. The kinetics of the transitions between these various states can be also estimated directly from MD trajectories and it is found very fast in this system, with a transition time of the order of ∼10^−1^ ns (Fig. 3d and S7b†).

Interestingly, decomposition of the FES into enthalpic (Δ*H*) and entropic contributions (−*T*Δ*S*) shows interestingly that the FES landscape (characterized by three minima almost equivalent in Δ*G*) is the result of a subtle interplay among the two terms (Fig. 3d). We observe that both enthalpy and entropy tend to favor lateral macrocycle configurations (Δ*H* and −*T*Δ*S* minima are found at |*d*| ≳ 0.4 nm). However, there is a clear mismatch between the position of the two red Δ*H* and blue −*T*Δ*S* minima: configurations where the macrocycle is localized close to the stoppers correspond to deep entropy minima (blue global minima at |*d*| ∼ 0.55 nm), while the enthalpically favored states (red Δ*H* global minima) are located at |*d*| ∼ 0.4 nm. This competition between enthalpy and entropy results in less pronounced Δ*G* wells. In particular, the lateral local Δ*G* minima correspond to intermediate lateral conformations for the macrocycle (|*d*| ∼ 0.45 nm) that are enthalpically unfavored and entropically favored (having positive Δ*H* and negative −*T*Δ*S* contributions). The situation is different in the global Δ*G* minimum at *d* = 0 nm, where the central configurations of the macrocycle appear to be enthalpically favored and entropically unfavored (enthalpy/entropy compensation,^72^ local red Δ*H* minimum *vs.* blue −*T*Δ*S* maximum at *d* = 0 nm).

While the entropically favored lateral macrocycle conformations are characterized by higher degrees of freedom and molecular mobility in the system,^73^ the central enthalpically-favored macrocycle configuration guarantees more favorable interactions in this system. In fact, monitoring the average number of HBs formed between the macrocycle and the formamidinium site at the center of the axle (Fig. 3e), we note that the HBs in the system reach a maximum when the macrocycle approaches the center of the axle (Fig. 3e, in pink: ∼1.7–1.8 HBs for |*d*| ≲ 0.25 nm). At the same time, calculation of the Solvent Accessible Surface Area (SASA) of this rotaxane as a function of *d* (Fig. 3e) shows two global SASA minima at |*d*| ∼ 0.45 nm, in correspondence of the two lateral Δ*G* minima. Together with the fact that such lateral conformations are entropically favored, these data indicate that the two lateral Δ*G* minimum conformations are controlled by solvophobic effects (*i.e.*, the macrocycle binds the stoppers reducing the interactions with the solvent). On the other hand, the central global minimum Δ*G* conformation is instead controlled by H-bonding.

Also in this case, our analysis on 2 provides detailed insights into the complex interplay between the various factors controlling the motion of the macrocycle (*e.g.*, solvent effects, conformational entropy, intermolecular interactions) that are often difficult to elucidate and typically precluded to the experiments.^66^ In particular, our analyses show how the fast dynamics of this formamidinium [2]rotaxane resembles what can be defined as a “Brownian flip-flop”^74^ between resonant configurations in a mechanically-interlocked molecule rather than the typical “rigid-like” diffusion process expected in a molecular shuttle.

### 2.4 Shuttling mechanism in a [10]CPP–fullerene [2]rotaxane

**Fig. 4:**
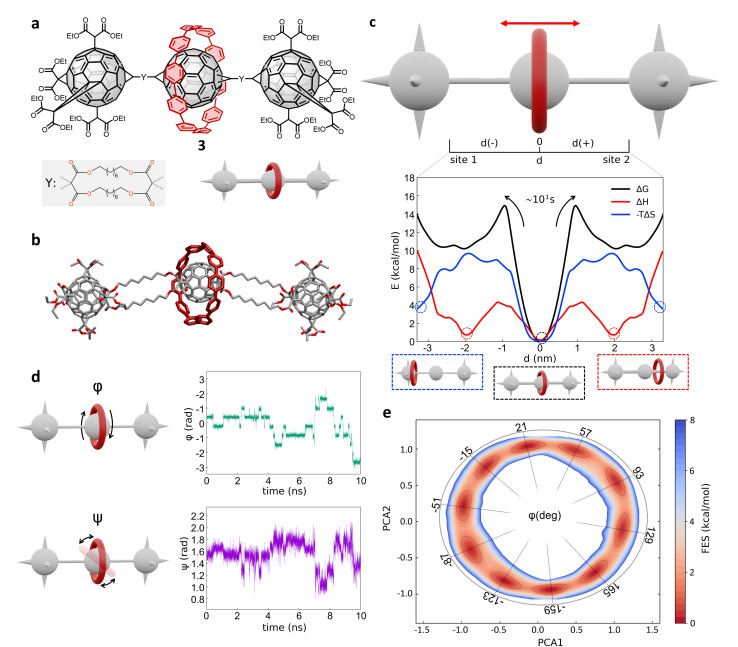
Shuttling mechanism and dynamics in [10]CPP–fullerene [2]rotaxane (3). (a) Structural chemical formula of [10]CPP–fullerene [2]rotaxane (3), Y: macrocyclic spacer and schematic representation of the rotaxane. (b) Atomistic molecular model of 3, hydrogen atoms are omitted for clarity. (c) Top: Schematic representation of the shuttling mechanism and of the translational CV *d* used in WT-MetaD simulations. Bottom: Free-energy profile (black), associated to the 3 system, with enthalpic (red) and entropic (blue) contributions. The boxes below show schematic representations of the relevant configurations, indicated by dashed circles on the free-energy profile. The arrows indicate the transition from the bound state (minimum) to the shuttling state, with the associated timescale. (d) Left: The two angles of macrocycle rotation around the central fullerene. The rotaxane is treated as cylindrically symmetric, where *ϕ* is the rotation around the *z*-axis and *ψ* is the tilting angle (rotation on the *XY* plane). Right: *ϕ* and *ψ* as a function of time, for a short portion of simulation. (e) Free-energy surface associated to the system in its bound state, as a function of CPP angular configurations (sin(*ϕ*), cos(*ϕ*) and sin(*ψ*), cos(*ψ*)) projected along the first two PCs. The *ϕ* angle corresponding to each minimum is reported (see ESI† for further details).

Next, we investigated a test system with a rigid macrocycle and a more flexible thread: a [2]rotaxane consisting of a [10]cycloparaphenylene ([10]CPP) that binds a central bis-adduct fullerene (C_60_) with two bulky fullerenes (C_60_) hexakis-adduct stoppers,^67^ separated by two long macrocyclic spacer groups (Fig. 4a: system 3). In the last decade, π-conjugated molecules, and in particular CPP–fullerene complexes^75–78^ have attracted great attention for their photophysical properties and potential applications. The rotaxane we studied here as the third case is characterized by a stable “bound” state, in which the CPP ring binds the fullerene, and “unbound” states in which the CPP ring lies on the spacer groups and moves toward the fullerene stoppers. While remarkable differences in the charge transport/recombination properties between those “bound” and “unbound” states have been shown in experiments,^67^ the relative dynamics of the system across these conformers remains elusive providing a convenient testing ground for our methods.

Following the same protocol employed for the previous cases, we built an AA model of *trans*-3 isomer of system 3 (ref. 67) that was then equilibrated *via* a preliminary MD simulation in explicit CHCl_3_ solvent (Fig. 4b). Starting from the resulting equilibrium structure, we performed a WT-MetaD simulation to explore the shuttling dynamics of the [10]CPP ring in rotaxane 3. To avoid spurious folding effects induced by the applied bias during the WT-MetaD simulation, the axle was kept extended during the simulation by softly restraining the position of the fullerene stoppers (see Methods in the ESI† for details). Such a constrained condition is consistent with the typical application of similar structures, which have been tested as molecular wires in charge transport components or as molecular junctions in a setup where the axle is connected to two electrodes.^79,80^ As in the case presented in Sections 2.1–2.2 (and later, Section 2.5), from a converged WT-MetaD simulation we could reconstruct the FES for the shuttling process, while multiple infrequent WT-MetaD simulations allowed us to reconstruct the height of the barriers and the characteristic kinetics for the involved transitions.

The FES in Fig. 4c (black line) shows the shuttling Δ*G* profile as a function of *d* (in black, see also Fig. S8† for details). The FES shows a deep Δ*G* minimum at *d* ∼ 0 nm, in which CPP is bound to the central fullerene (“bound” state). The time required to unbind and leave the central fullerene, estimated *via* infrequent WT-MetaD simulations, is found in the order of tens of seconds. It is informative to compare such a transition time to the typical escape time of the CPP ring from a [10]CPP–pseudorotaxane system to the solution. In such an asymmetric variant of this specific system, the C_60_ has only a single adduct, so that the CPP ring can be effectively released in solution directly from the bound state (see ESI, Fig. S9a,† top). The kinetics of such a ring unbinding transition has been estimated experimentally to occur in the timescale of milliseconds.^67^ Infrequent WT-MetaD simulations activating the ring unbinding from such an asymmetric system variant provided a comparable unbinding timescale, within the error that can be expected from such approaches (see Fig. S9 and Methods section in the ESI† for details). On the one hand, such an agreement demonstrates the accuracy of the AA models and the appropriateness of the simulation setup adopted herein. On the other hand, this indicates that a direct ring unbinding event from a terminal fullerene group is orders of magnitude faster than the characteristic time required for the same macrocycle to leave the binding from the central fullerene and to diffuse along the axle in rotaxane 3 ∼ (2.5 orders of magnitudes faster if comparing the unbinding times computed *via* infrequent WT-MetaD, Fig. S9b†). This indicates a possible frustration of the CPP unbinding and diffusion processes in this rotaxane where, *e.g.*, the ring can unbind and move from the fullerene only following specific directions determined by the substituents and the axle orientations (see bent axle orientation in, *e.g.*, Fig. 4b). This reduces the accessible pathways toward unbinding and shuttling along the flexible linkers, which has a significant effect on the kinetics of such a transition.

A decomposition of the FES into Δ*H* and −*T*Δ*S* contributions demonstrates that the global Δ*G* minimum in this system (corresponding to the macrocycle in central, bound configuration) is both enthalpically and entropically favored (see Fig. 4c, bottom: black, blue and red curves minima at *d* = 0). This configuration allows maximizing the π stacking interaction between [10]CPP and the central C_60_ fullerene, while at the same time preserving a high entropy in the system. In this bound state, the macrocycle can in fact easily rotate on the central fullerene, which guarantees a high number of degrees of freedom: this state thus corresponds to multiple possible rotationally-symmetric conformations for the ring on C_60_ that are identical in energy while the macrocycle can “freely” rotate along the main axis of the dumbbell. We believe that in this respect, the match between the rigid [10]CPP macrocycle and the rigid C60-bisadduct binding site is rather unique.

Coupled to the translational motion of the macrocycle, such a rotational degree of freedom of the macrocycle around the central fullerene and the main rotaxane axle demonstrates how this rotaxane exhibits multi-layered, complex dynamics characterized by multiple degrees of freedom and motion types. The translational motion of the macrocycle is accompanied by an entropic cost^81^ that is required to have the macrocycle to pass through the flexible linkers (already observed in similar [2]rotaxanes with flexible linkers^28,82^). The possible conformations of the linkers are strongly reduced by the rigid ring encircling them and the diffusion of the ring is directionally constrained by the axle. In fact, the blue −*T*Δ*S* profile of Fig. 4c (bottom) shows that configurations where the macrocycle is diffusing along the flexible axle are entropically unfavorable. At the same time, such an entropic cost is partially compensated by the increased interaction between CH_2_ groups of the linkers and CPP in an enthalpy–entropy compensation mechanism,^72^ consistent with what also seen in ROESY NMR experiments^67^ (see ESI Movie 2,† showing translational motion of the macrocycle and the interaction with the spacers). This reflects in the local minima in the red Δ*H* profile of Fig. 4c at |*d*| ∼ 2 nm, which are in very good agreement with the observation of an “unbound state” between CPP and C60 during ultrafast pump–probe spectroscopy.^67^

To obtain deeper details on the dynamics of the [10]CPP macrocycle in the bound state around the central fullerene, we also performed 1 μs unbiased MD simulation starting from this configuration. We decomposed the motion of [10]CPP around the fullerene in terms of macrocycle rotation (*ϕ*, Fig. 4d-top panel) and tilting (*ψ*, bottom panel). The rotation can assume values between −π and π radians while the tilting ranges between π/4 and 3/4π radians. To better decompose and visualize this angular ring motion we collected the *ϕ* and *ψ* sines and cosines data, and operated a principal components (PC) analysis over these four variables. As expected from a quasi-2D motion, the first two PCs contain the majority of the fluctuations (∼99%). In Fig. 4e we show the projection of the sampled ring conformations onto the first two PCs. The density of the points in given positions of this plot (distribution) provides information on the most probable macrocycle positions/motions. From the density plot, we could reconstruct the associated FES *via* histogram conversion (see Fig. 4e), where all configurations of the CPP macrocycle (when bound around the C_60_) are associated to their Δ*G*. This FES exhibits 10 energetically equivalent minima arranged in a circle. These states are associated with different values of the rotation angle *ϕ* of CPP around the fullerene, while the width of the distribution around the circle is associated with the tilting motion (see also Fig. S10†). Each minimum is separated by a free-energy barrier of ∼2 kcal mol^−1^. Interestingly, this result is in remarkable agreement with the energy barrier associated to the dynamic rotation of fullerene inside a carbon nanohoop, as reported by Matsuno *et al.*^83^

The presence of ten clear periodic minima indicates that the motion is step-wise, determined by the possible π-stacking of [10]CPP and fullerene benzene rings. We also employed a clustering analysis (probabilistic analysis of molecular motifs^84^) to capture the transitions between these angular minima along the MD simulations, which allowed us to characterize the kinetics of the step-by-step CPP rotation around the fullerene. We thus estimated a mean residence time for the CPP in each minimum configuration as ∼0.1 ns, which reveals that such step-wise macrocycle rotation is fast in these conditions. This combination of PC and clustering analysis provides a further, useful strategy to characterize the complex dynamics in these systems, providing in this case a quantitative proof of the high-rotational degrees of freedom of the macrocycle in this bound state, and demonstrating how such bound state correspond to a highly entropically favored minimum energy configuration (Fig. 4e: deep black and blue minima at *d* = 0 nm).

The proposed AA simulations study exhaustively characterized both translational and rotational degrees of freedom of system 3, providing a thermodynamic picture consistent with the experimental observations,^67^ and atomistic details that are difficult to attain experimentally. This enriches this study with a different test case with respect to the previously explored ones, and at the same time demonstrates the flexibility of the adopted approach.

### 2.5 Shuttling mechanism in a rigid bistable [2]rotaxane

**Fig. 5:**
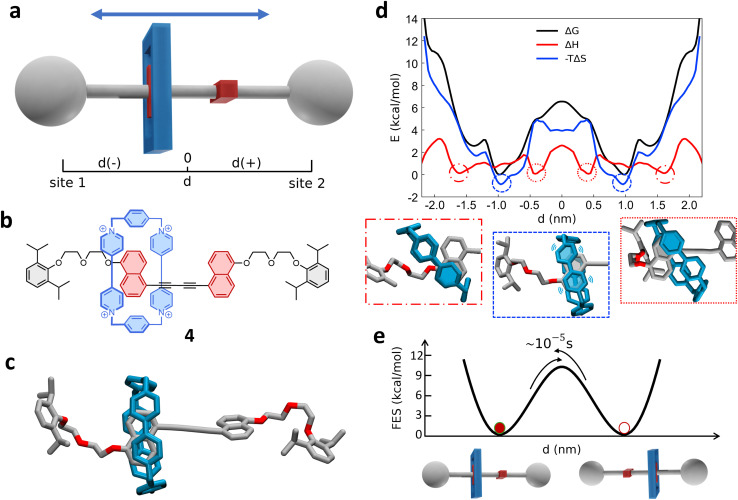
Shuttling dynamics of rigid bistable [2]rotaxane (4). (a) Schematic representation of the shuttling mechanism and the translational CV *d* used in WT-MetaD simulations. (b) Structural chemical formula and (c) atomistic molecular model of rigid bistable [2]rotaxane (4). (d) Top: Free-energy profile (black), associated with the 4 system, with enthalpic (red) and entropic (blue) contributions. Bottom: Insets showing representative snapshots of three relevant states (indicated in the plot by dashed, dotted and dot-dashed circles) explaining the details of the shuttling mechanisms. (e) Diagram showing the two free-energy minima in which the blue-box is located at the two recognition sites. The arrow indicates the transition of the blue-box between the two recognition sites, with the associated transition time.

As a final example, we also investigate a degenerate bistable [2]rotaxane. This consists of a dumbbell containing two naphthalene (NP) recognition sites and the tetracationic macrocycle cyclobis(paraquat-*p*-phenylene) (CBPQT^4+^), the so-called “blue-box” (Fig. 5).^28^ From now on, we will refer to this system as 4. In this architecture, the rigid spacer between the two recognition sites determines a simpler (more rigid-like) dynamics compared with more flexible systems. Nonetheless, a complete comprehension of the on/off mechanisms and of the internal dynamics in this system is non-trivial to attain.

We developed an AA model of 4 (Fig. 5c, see Methods in the ESI† for details), that was neutralized with PF_6_^−^ counterions, immersed and equilibrated in explicit acetone solvent, to reproduce experimental conditions.^28^ As in the previous cases, we then employed WT-MetaD simulation to explore the shuttling mechanism in the rotaxane, obtaining the free-energy landscape (FES) as a function of the translational position *d* of the blue-box macrocycle along the axle (Fig. 5d and S11†). The computed FES demonstrates a sharply “discrete I-0” shuttling mechanism in this system, in which the blue-box switches between two states corresponding to the binding of the cycle onto the two symmetric recognition sites (stations). The blue-box binding to the stations is rather strong, and the shuttling from one station to the other requires crossing a high activation barrier.^28^

The black FES profile of Fig. 5d (obtained from WT-MetaD) preliminarily indicates an underestimated value for the latter of at least ∼7 kcal mol^−1^ (black barrier at *d* = 0 nm). From infrequent WT-MetaD simulations the Δ*G*^‡^ has been estimated to be around ∼10–11 kcal mol^−1^ (Fig. 5e) – in better agreement with the experimentally estimated value of ∼9.7 kcal mol^−1^ (see also ESI† for further details)^28^ – and a characteristic transition/unbinding timescale in the range of ∼10^−1^ seconds. It is worth noting how, also in this case (and as in the cases of Fig. 1, 2 and 4), the presence of such considerable transition barriers requires an enhanced sampling approach to effectively study such transitions at such atomistic resolution. The adopted WT-MetaD approach demonstrates flexibility and generality, and is found to effectively work the purpose.

Enthalpic and entropic contributions of the free-energy in Fig. 5d show clearly how the global minima of the FES (minima in the black Δ*G* profile at *d* ∼ ±1.0 nm) are mostly entropically driven, since they also correspond to the entropic minima (blue dashed circle in the blue −*T*Δ*S* profile and blue dashed box below). On the other hand, four main enthalpy minima have different locations along the axis of 4 (circled in the red Δ*H* profile). A deeper inspection of the configurations corresponding to these entropic and enthalpic minima (Fig. 5d: bottom insets) elucidates the nature of these states and reveals the details of the shuttling mechanism in this rotaxane. During the shuttling of the diffusing blue-box along the axle, the CBPQT^4+^ gets in contact with the NP groups. When this happens, the rings of the two components start to interact. Hypothesizing, for example, a CBPQT^4+^ movement left-to-right along the axle, the enthalpy minima correspond to configurations in which a first pyridine ring of CBPQT^4+^ stacks onto one of the axle NP rings (see, *e.g.*, the first snapshot on the left in Fig. 5d, bottom). This produces a local macrocycle–axle interaction increase and a favorable enthalpic change (first Δ*H* minimum at *d* ∼ −1.6 nm: a symmetrical minimum is found on the other side of the rotaxane). Proceeding left-to-right, the blue-box surrounds the NP rings: this is where we find the minimum of the free-energy. In contrast, such free-energy minima correspond to a more dynamical state, in which the bipyridine group of the blue-box fluctuates next to the NP without a well-defined stacking of the two aromatic rings. Such a “dynamic/fluctuating binding” allows preserving to some extent the ring–ring interactions while at the same time guaranteeing high mobility and preserving the degrees of freedom of the blue-box. From this originates the entropic stabilization of these minimum free-energy states. Moving further left-to-right, a high entropic penalty thus accompanies the motion of CBPQT^4+^ when this surpasses the NP recognition states (|*d*| < 0.6 nm). At *d* ∼ −0.4 nm another Δ*H* minimum is encountered, very similar to that previously seen at *d* ∼ −1.6, when the blue-box, leaving the NP, preserves its interaction with it *via* a one-ring stacking with it (Fig. 5e: bottom inset on the right). It is interesting to note also how the establishment of such ring–ring stacking corresponds to a minimum in enthalpy and at the same time to an unfavorable barrier in entropy (interaction *vs.* blocking). The high shuttling free-energy barrier starting at *d* ≳ −0.6 nm in Fig. 5d (in black) is thus first due to an entropic barrier (in blue) and then, upon breakage of the last ring–ring CBPQT^4+^–NP interaction, also by an enthalpic one – see the similar shape of the black and red barriers profiles for *d* ≳ −0.25 nm in Fig. 5d (all profiles have then symmetrical feature respect to *d* = 0).

Infrequent WT-MetaD simulations provide also more detail into the kinetic characterization of blue-box movement between the two main metastable states, namely the dynamic binding with recognition sites (see scheme in Fig. 5e and S12†). For steric reasons, the NP site plane needs to assume a parallel configuration respect to the CBPQT^4+^ bipyridine plane in order to allow the threading of the macrocycle along the axle and the binding with the other recognition site. Provided the entropic nature of free-energy minima seen in Fig. 5e, as said we can impute such a kinetic barrier of transition from one NP site to the other mostly to this constraint. It is also interesting to note that in order to move from one NP site to the other one, the CBPQT^4+^ macrocycle has to rotate, due to the quasi-orthogonal conformation of the two NP groups. This provokes steric contacts and impacts before crossing the intermediate axle point (*d* = 0 nm), which fit on a molecular level with the entropy and enthalpy penalties that accompany the shuttling.

Overall, also in this more challenging case, such a simulation approach demonstrates its flexibility, providing results in optimal agreement with the available experimental evidence^28^ and atomistic details that are difficult to attain with other approaches. Interestingly, these simulations show that the streamlined, bistable dynamics of this system result from subtle entropic effects and from their modulation with enthalpic ones, which need to be understood to rationally regulate the switching kinetics of the rotaxane.

## 3 Conclusions

Mechanically interlocked molecules like rotaxanes are important platforms to construct molecular machines and are attracting great interest. However, understanding the molecular-level principles that control their dynamic behavior is often not trivial and experimental approaches based on NMR spectroscopy only shine light on a small part of a complex picture. In the present work, we describe a comprehensive *in silico* approach that allows investigating the dynamics of MIMs at submolecular resolution. By means of AA molecular modeling and enhanced sampling techniques based on standard and infrequent WT-MetaD we explored the translational motion and dynamics of four MIMs, differing in their architecture, structural and dynamical features.

Our investigation provided results in excellent agreement with the experimental evidence available for the studied systems. From our simulations, we gained deep molecular-level insights into the free-energy characterization of such MIMs, enabling us to determine the most favorable states that the interlocked molecules populate at equilibrium and the key interactions that control the systems. At the same time, the kinetic characterization of the transitions connecting the different metastable states provided detailed information on rate limiting steps and pathways that rule the shuttling in these systems.

Decomposition of the free-energy profiles into the enthalpic and entropic components revealed how a delicate balance between various intermolecular interactions, *e.g.*, H-bonding, solvent effects, and conformational entropy determine the dynamical behavior of such molecular machines.

In particular, the results presented here demonstrate how the behavior of MIMs is not only dictated by their chemical structure, but also by their interaction with an external environment that can play an active role, and how changing environmental variables such as the solvent type, can have non-trivial (*e.g.*, local and non-averageable) effects. In this sense, the results reported in Fig. 2 show how thinking of a rotaxane as a structural object immersed in a solvent that acts as a “field” may be misleading in some cases while on the contrary, it may be more appropriate to think of rotaxane-plus-solvent as a single, complex molecular system.

Given the considerable effort made by the community in recent years, which offer a much wider range of systems than the four explored here, we underline that the scope of this work is to demonstrate the versatility of this approach, which can be in principle applied to various other MIMs.

We believe that the comprehensive thermodynamic/kinetic characterization that can be attained with such computational approaches will be important in the context of MIMs, where a deep understanding of the key factors controlling their structural and dynamical properties is fundamental for the engineering of molecular motors, switches, and molecular machines.

## Author contributions

G. M. P. conceived this research and supervised the work. L. L. developed the molecular models and performed the simulations. L. L. performed the analysis, with the help of L. P., M. S., C. P. and G. M. P. L. L., C. P., L. P., M. S., M. v. D. and G. M. P. discussed the results and contributed to the writing of the manuscript.

## Conflicts of interest

There are no conflicts to declare.

## Supplementary Material

SC-014-D3SC01593A-s001

SC-014-D3SC01593A-s002

SC-014-D3SC01593A-s003

## Data Availability

Details on the procedures for the parametrization of the molecular models and on the simulations' setup, along with additional simulation data, are provided in the Methods section and in the ESI† file. Complete data and materials pertaining to the molecular simulations conducted herein (input files, model files, raw data, analysis, *etc.*) are available at: https://zenodo.org/record/7991733#.ZHePX6VByUn (DOI: https://doi.org/10.5281/zenodo.7991733). All the PLUMED input files required to reproduce the results reported in this paper are also available on PLUMED-NEST (https://www.plumed-nest.org), the public repository of the PLUMED consortium, as plumID:23.021. Other information needed is available from the corresponding author upon reasonable request.
