# Supplementary material for: Into the dynamics of rotaxanes at atomistic resolution
Source: Chem Sci. 2023 May 30;14(24):6716–29. doi: 10.1039/d3sc01593a (PMC10283497; doi:10.1039/d3sc01593a)
Supplement: SC-014-D3SC01593A-s001 [file SC-014-D3SC01593A-s001.pdf]

Supplementary Information (SI) for Chemical Science.
This journal is © The Royal Society of Chemistry 2026

Supplementary Information for:

Into the Dynamics of Rotaxanes at Atomistic Resolution

Luigi Leanza,[†] Claudio Perego,[‡] Luca Pesce,[‡] Matteo Salvalaglio,[¶] Max von Delius,[§] and Giovanni M. Pavan[*,†,‡]

†*Department of Applied Science and Technology, Politecnico di Torino, Corso Duca degli Abruzzi, 24, I-10129 Torino, Italy*

‡*Department of Innovative Technologies, University of Applied Sciences and Arts of Southern Switzerland, Polo Universitario Lugano, Campus Est, Via la Santa 1, 6962 Lugano-Viganello, Switzerland*

¶*Department of Chemical Engineering, University College London,London WC1E 7JE, United Kingdom*

§*Institute of Organic Chemistry, Ulm University, Albert-Einstein-Allee 11, 89081 Ulm, Germany*

E-mail: giovanni.pavan@polito.it

This version of the Supplementary Information was published online on 22nd July 2026, clarifying some of the computational details and adding the link to the permanent repository of files on Zenodo

Computational Details

Simulation Setup

All the model systems and ions were parametrized using the Generalized Amber Force Field (GAFF)[1] (the β dihedral angle of **2** has been futher optimized via dihedral scan to best

fit the torsional energy barrier estimated via DFT calculations). The partial charges for the atomistic models have been calculated using the RESP[2] method, computed at Hartree-Fock (HF) level of theory, with 6-31G basis set, as implemented in Gaussian16.[3] Only in the case of the $CBPQT^{4+}$ macrocycle of Fig. 5 in the main paper, the AM1-BCC method[4,5] was used, as it was providing partial charges that seemed more correct. AmberTools21[6] package was used for the parametrization of the systems. The solvents parameters were obtained from the *virtualchemistry.org* database.[7,8] A proper amount of PF_6^- ions have been added to neutralize the total charge of the systems. All the systems simulated herein have been minimized via steepest-descent and equilibrated in NPT condition (constant N: number of particles, P: pressure, T: temperature). Production runs were conducted in the NPT ensemble for up to 2 μs. Temperature and pressure are kept constant (298 K and 1 bar), using the v-rescale thermostat[9] and the Berendsen,[10] and Parrinello-Rahman[11] barostats (see Table S1 for details). For each system we have chosen the barostat algorithm allowing the most efficient system equilibration using 2 fs timesteps (i.e., reaching stable equilibrium MD in a convenient simulation time). Long-range electrostatic interactions have been treated using the Particle-Mesh-Ewald (PME)[12] and a cutoff of 1.0 nm. Van der Waals interactions were truncated at 1.0 nm. The LINCS algorithm[13] has been used to constrain all the bonds involving hydrogens. The leapfrog integrator was used to propagate dynamics with a time step of 2 fs.
All the simulations were carried out using GROMACS[14] 2021.6, patched with PLUMED,[15] version 2.7.[16]

Complete details on the molecular models developed herein and input files for simulations conducted herein are available in the Zenodo folder https://zenodo.org/records/7991733 (DOI: https://doi.org/10.5281/zenodo.7991733). All the PLUMED input files required to reproduce the results reported in this paper are also available on PLUMED-NEST (https://www.plumed-nest.org), the public repository of the PLUMED consortium, as plumID:23.021.

Switching rates estimation via infrequent Well-Tempered Metadynamics

Switching rates are typically rare events at the timescale accessible to classical all-atom simulations. It has been demonstrated that the kinetics of rare events can be properly reconstructed using infrequent Well-Tempered Metadynamics (WT-MetaD).[17,18] With an opportune choice of the collective variables (CVs) and setup of the WT-MetaD,[18] the real transition time t from basin A to B can be calculated from a biased WT-MetaD trajectory as:

$$t = t_{WT-MetaD} \langle e^{\beta(V(s(\mathbf{R}),t))} \rangle_{WT-MetaD} \tag{S1}$$

where s($\mathbf{R}$) is the CV, V(s($\mathbf{R}$), t) is the time-dependent bias, β is the inverse temperature, and the exponential is averaged over the WT-MetaD run.

For the approach to work, it is essential that V(s($\mathbf{R}$), t) is added infrequently to minimize the probability to add bias in portions of the CV space corresponding to the transition region.

From multiple infrequent WT-MetaD runs, one can build an empirical cumulative distribution function (ECDF) that must fit well with the Poisson distribution expected for rare events:[19]

$$P_{n\geq 1} = 1 - P_0 = 1 - e^{\frac{t}{\tau}} \tag{S2}$$

where τ is the characteristic timescale of the transition.

The kinetic rate constant (k) associated with the rare events can be estimated as the inverse of the characteristic timescale τ:

$$k = \frac{1}{\tau} \tag{S3}$$

The barrier associated to the transition $\Delta G^{\ddagger}$ can be calculated from the Eyring equation:

$$k = \frac{\kappa K_B T}{h} e^{\frac{-\Delta G^{\ddagger}}{RT}} \tag{S4}$$

where κ is a transmission coefficient (equal to one in the no-recrossing assumption of transition state theory), K_B is Boltzmann's constant and h is Planck's constant.

To quantitatively compare the theoretical and the empirical distribution, the Kolmogorov-Smirnov (KS) test[20,21] has been performed.

System-specific simulation protocols.

We will here discuss the choice of CVs and biasing approach adopted to study the shuttling dynamics in each of the studied systems. We underline that, for each case, WT-MetaD parameters have been optimized to improve the convergence of free-energy differences and kinetic quantities extracted for all the studied systems. For example, in WT-metaD simulations we have adjusted the bias factor according to the characteristic free-energy differences associated with the shuttling of each system. In the case of infrequent WT-metaD, we have also modulated the deposition rate and initial height of the deposited Gaussians in order to maximize the P-value of the KS test.[18]

Shuttling and kinetics of $[R_4 - H_2]^{2+}$ **[2]Rotaxane**

To study the translational dynamics of system **1**, we employed WT-MetaD simulations, biasing: 1) the difference d of the distance between the center of geometry of the ring and the center of geometry of one benzimidazolium (d1) and between the center of geometry of the ring and the second benzimidazolium (d2); 2) the distance $d_{benz-benz}$ between the two benzene rings of the dibenzo[24]crown-8 ether macrocycle (see the schematic representation of CVs in Fig.S1). The bias has been updated every 500 time steps (1 ps of simulation time) using Gaussians of initial height 1 kJ/mol, the width of 0.1 nm and 0.05 nm for CV1 and CV2, respectively, and a bias factor of 20 in DMSO, 30 in ACN, and 40 in DCM and $CHCl_3$. For each system, we ran a minimum of 1.5 μs, allowing us to record multiple shuttling events across stations. For the calculation of the shuttling rates in **1** from bound to unbound we ran 25 infrequent WT-MetaD simulations, starting from the bounded state and biasing the

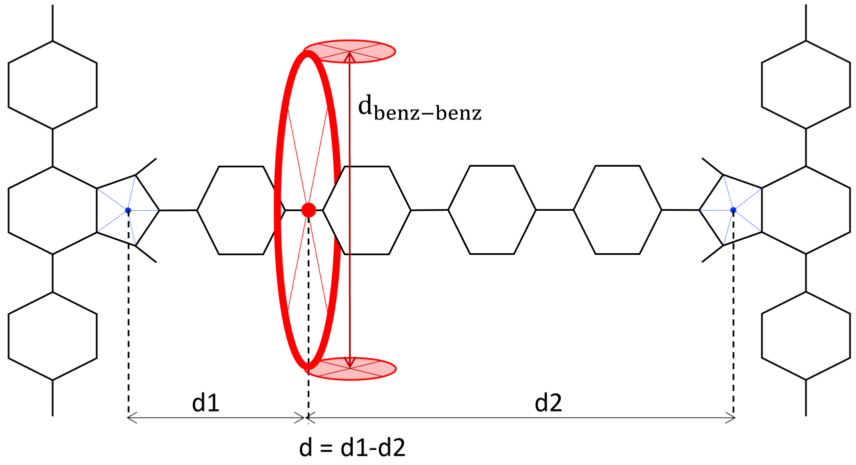

Figure S1: Schematic representation of WT-MetaD simulation setup for system **1**. The two selected CVs d and $d_{benz-benz}$ are displayed.

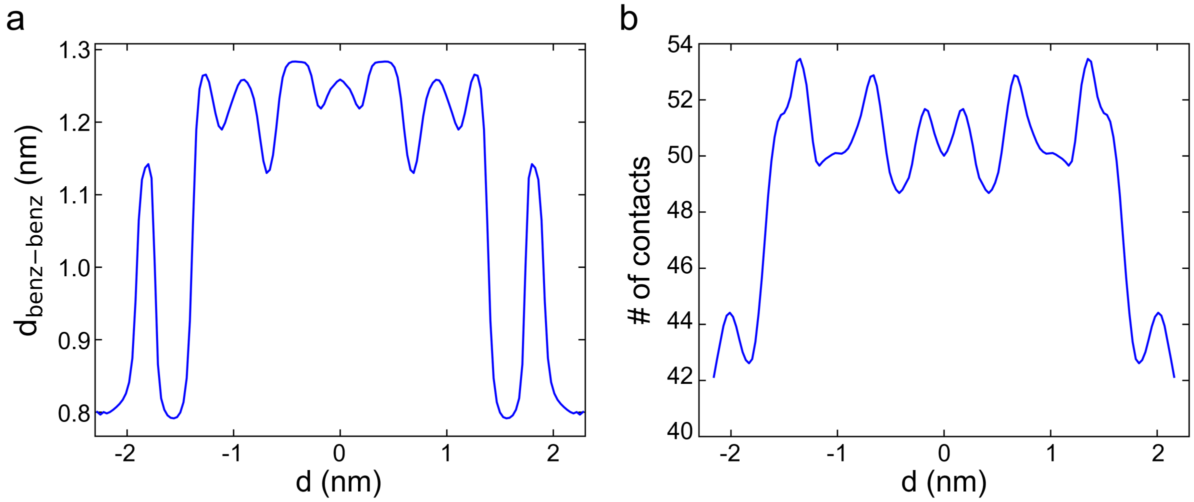

Figure S2: (a) Mapping of $d_{benz-benz}$ along d. (b) Mapping of the number of contacts between the axle and the ring along d. The mapping are obtained using the approach of Ref. 22.

distance between the center of geometry of the ring and the center of geometry of the closest benzimidazolium (*d1*, CV1) and the distance ($d_{benz-benz}$, CV2) between the two benzene rings of the dibenzo[24]crown-8 ether macrocycle. Each run ends when CV1 falls in basin D (see 2D-FES in Fig. 1d of the main paper). The resulting ECDF and the fitting with the Poissonian distribution is showed in Fig. S3a. In **ACN**, the bias has been added every 20 ps using Gaussians of initial height 1 kJ/mol, width of 0.1 nm and 0.05 nm for CV1 and CV2,

respectively, and a bias factor of 20. From the WT-MetaD simulation of system **1** in ACN, we mapped the $d_{benz-benz}$ and the number of contacts between the axle and the ring along the variable d (Fig. S2), as proposed in Ref. 22.

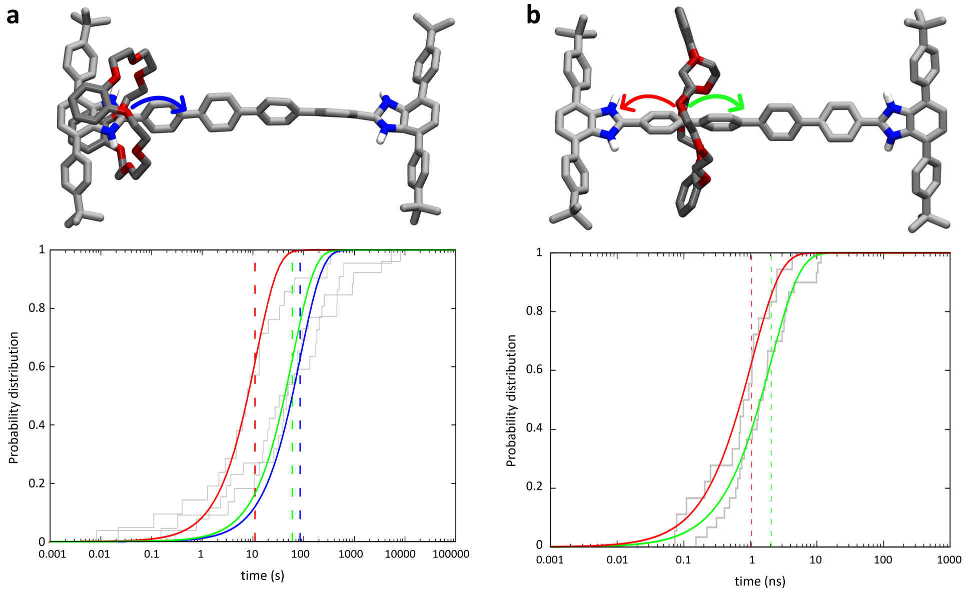

Figure S3: (a) Top panel: atomistic molecular structure of **1**, state A. The blue arrow indicates the transition to state D. Bottom panel: Transition times estimated via multiple infrequent metadynamics simulations starting from states A (red curve), B (blue), and C (green) and reaching state D. The ECDF of the measured transition times are fitted by Poissonian probability distributions (continuous curves). (b) Top panel: atomistic molecular structure of **1**, state D. The red and green arrows indicate the two possible directions of motion of the macrocycle. Bottom: Transition times estimated via multiple MD simulations (gray steps) and Poissonian probability distributions (red and green curves) for the D→A and D→D' motion of the macrocycle along the dumbbell. The vertical dashed red and green lines represent the characteristic timescale τ associated with the events and are estimated by fitting the ECDF with the Poissonian distributions.

In **$CHCl_3$** and **DCM**, the bias has been added every 50 ps, using Gaussians of initial height 2 kJ/mol, a width of 0.1 nm and 0.05 nm for CV1 and CV2, respectively, and a bias factor of 45. In **DMSO**, the bias has been added every 80 ps, using Gaussians of initial height 2.5 kJ/mol, width of 0.1 nm and 0.05 nm for CV1 and CV2, respectively, and a bias factor of 25.

The times for transitions D $\rightarrow$ A and D $\rightarrow$ D' in ACN are collected via multiple classical MD

runs. Every 500 simulation steps, PLUMED checks whether CV1 is in basin A or D' and, eventually, stops the simulation. The ECDFs and the fitting with the Poissonian distribution are displayed in Fig. S3b.

Fig. S5 shows the FESs as a function of the two CVs biased during the WT-MetaD simulations in all the for solvents investigated in this study. Fig. S6 shows the height of the barriers of unbinding from the recognition site in the four solvents, estimated via infrequent metadynamics simulations.

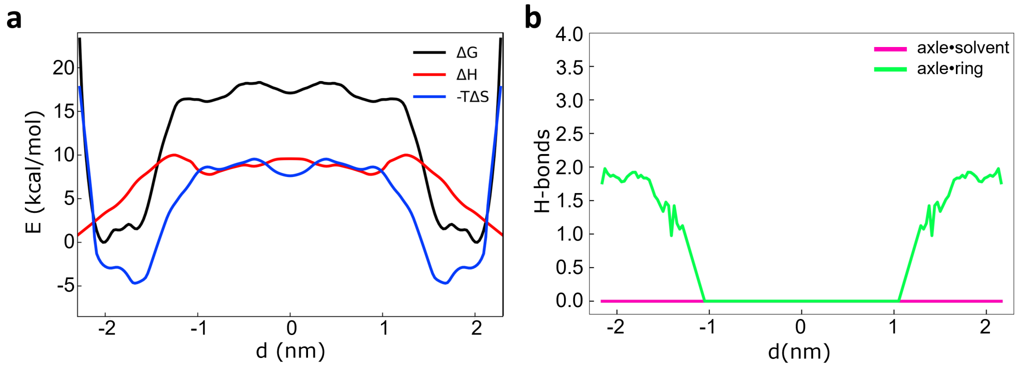

Figure S4: On the left, free-energy (black), enthalpy (red) and entropy (blue) profiles of the **1** immersed in DCM solvent ($\beta = 0.10$) as a function of d. On the right, number of HBs between the axle and the macrocycle (green) and between the axle and the solvent molecules (purple line), as a function of d.

Shuttling and kinetics of formamidinium [2]Rotaxane

In system **2**, we run 1μs of classical MD simulation, in NPT conditions. The 2D-FES has been computed along the difference between the distance between the center of geometry of the macrocycle and the center of geometry of one stopper and the distance between the center of geometry of the macrocycle and the second stopper (d), as showed in Fig. S7a. ECDF obtained via multiple MD simulations and Poissonian probability distributions for the shuttling motion of the ether crown of **2** are showed in Fig. S7b.

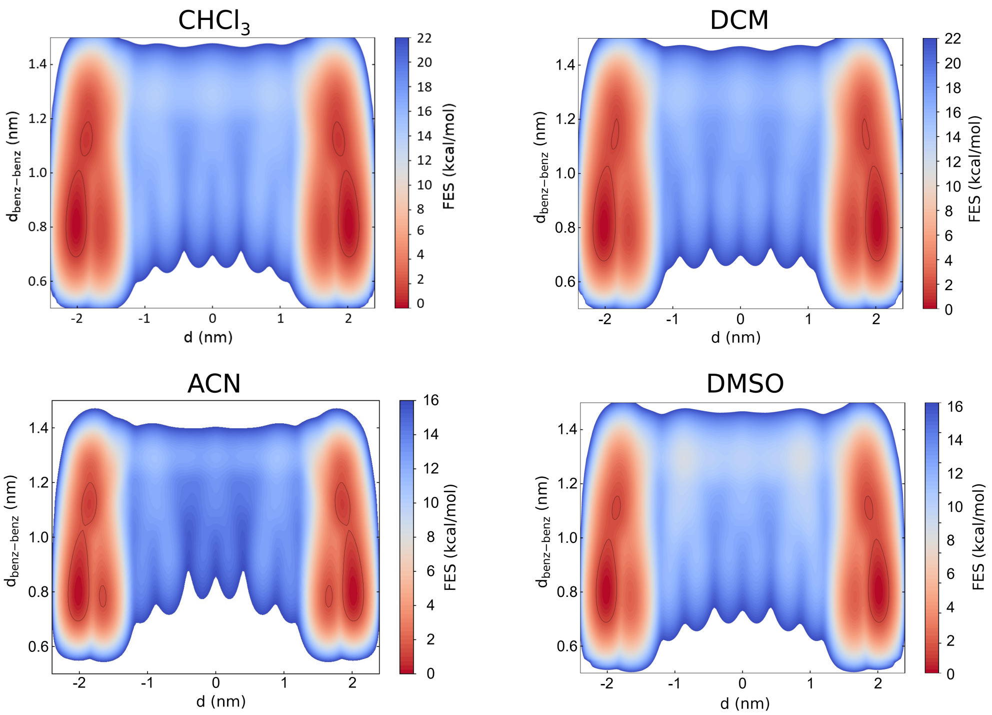

Figure S5: FESs of **1** as a function of the two CVs biased during WT-MetaD simulations in all the four solvents investigated in this study. The free-energy profiles are symmetrized with respect to $d = 0$, exploiting the symmetry of the system.

Shuttling and kinetics of [10]CPP Fullerene Pseudorotaxane and [10]CPP Fullerene [2]Rotaxane

The rates for the decomplexation of [10]CPP-Fullerene Pseudorotaxane[23] have been calculated running 20 infrequent WT-MetaD using the distance between the center of geometry of [10]CPP and the center of geometry of the fullerene as CV, depositing Gaussians every 40 ps, 0.5 kJ/mol of initial height, 0.1 nm width and a bias factor of 10. The obtained ECDF and the fitting with the Poissonian distribution for [10]CPP-Fullerene Pseudorotaxane is showed in Fig. S9a.

For system **3**, we used a WT-MetaD bias acting on the difference d between the distance between the center of geometry of the [10]CPP and the center of geometry of one stopper (d1) and the distance between the center of geometry of the [10]CPP and the second stopper (d2); Gaussians have been added every ps, with an initial height of 1 kJ/mol, gaussian width

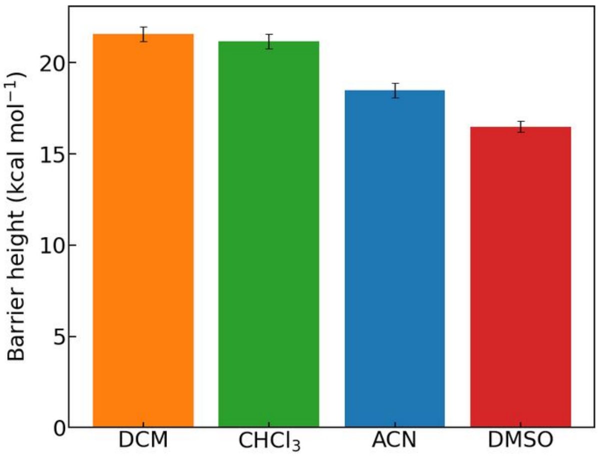

Figure S6: Barrier heights of unbinding from the recognition site of **1** in various solvents, estimated from infrequent metadynamics.

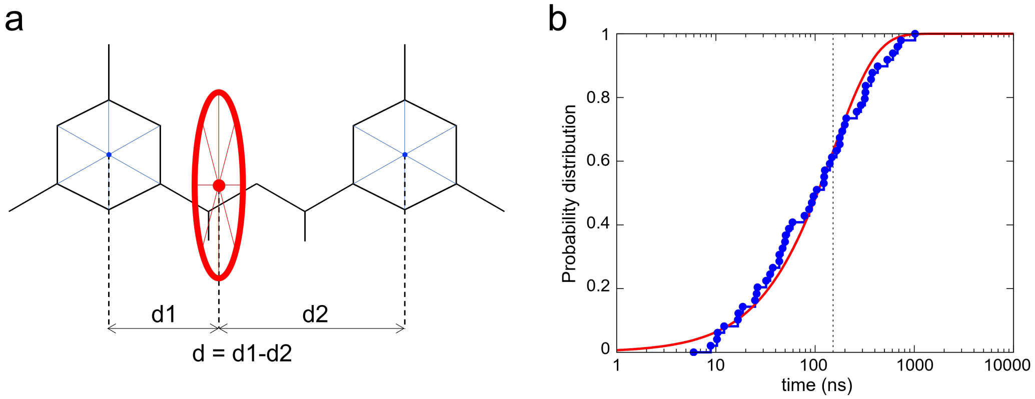

Figure S7: (a) Schematic representation of **2**. The CV d used for reweighting the free-energy surfaces is displayed. (b) Transition times are estimated via multiple MD simulations (blue steps) and Poissonian probability distributions (red curve) for the shuttling motion of the ether crown of **2**. The vertical dashed gray lines represent the characteristic timescale τ associated with the event, estimated from fitting the ECDF with a Poissonian distribution.

of 0.3 nm, a bias factor of 25. To prevent the *trans*-3 fullerene bisadduct from closing under the effect of the metadynamics bias, we imposed an external wall potential that acted on the center of geometry of the two fullerene hexakis-adduct stoppers when their displacement from reference is greater than 1 nm, with a force constant equal to 200 (kJ/mol)/nm^2. For a quantitative estimation of the characteristic timescale associated with a transition from "bound" to "unbound" states in **3**, three CVs were used: 1) The distance between the center of geometry of [10]CPP and the center of geometry of the central fullerene (CV1); 2)

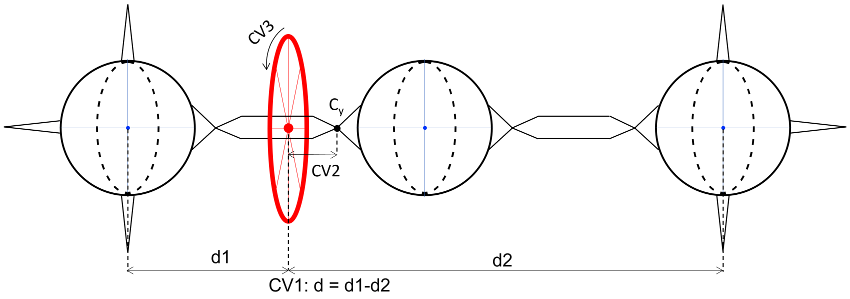

Figure S8: Schematic representation of WT-MetaD simulation setup for system **3**. The selected CVs (CV1, CV2, CV3) are displayed.

The distance between the center of geometry of [10]CPP and the first carbon atom of the substituent C_y (CV2); 3) The relative rotation between [10]CPP and the central fullerene (CV3). A schematic representation of the CVs used for the WT-MetaD simulation of **3** is showed in Fig. S8. The bias has been added every 40 ps using Gaussians of initial height 0.5 kJ/mol, width of 0.1 nm for CV1 and CV2 and 0.3 radians for CV3, and a bias factor of 10. The obtained ECDF and the fitting with the Poissonian distribution for **3** is showed in Fig. S9b.

In Fig. S10, we show the free-energy profile of CPP rotation in the polar plane having ϕ as angular phase and ψ as magnitude of the vector. While this representation is more straightforward than the PCA-based plot in Fig. 4e of the main manuscript, and successfully reproduces the periodicity features of the CPP rotation, it distorts the appearance of the free-energy minima associated to the different ϕ angles. Instead, the usage of PCA allows to conveniently represent the free-energy basins associated to different rotation angles ϕ as symmetric (see Fig. 4e).

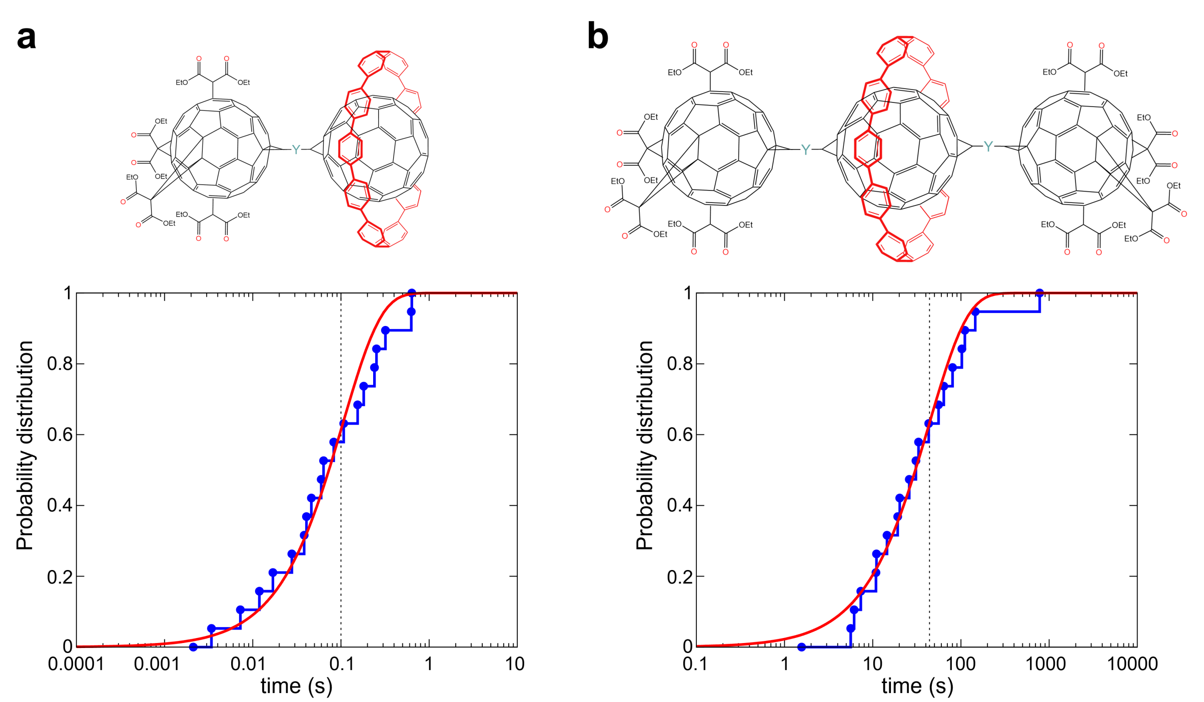

Figure S9: (a) Transition times for [10]CPP Fullerene Pseudorotaxane decomplexation (jump in solution) estimated via multiple infrequent WT-MetaD (blue steps), and Poissonian probability distributions (red curve). (b) Estimated times in **3** for [10]CPP to leave the central Fullerene ("bound" state) and jump on one lateral stopper ("unbound" state), collected via multiple infrequent WT-MetaD (blue steps), and Poissonian probability distributions (red curve). In both panels, the vertical dashed lines represent the characteristic timescale τ associated with the events and are estimated by fitting the ECDF with the Poissonian distributions.

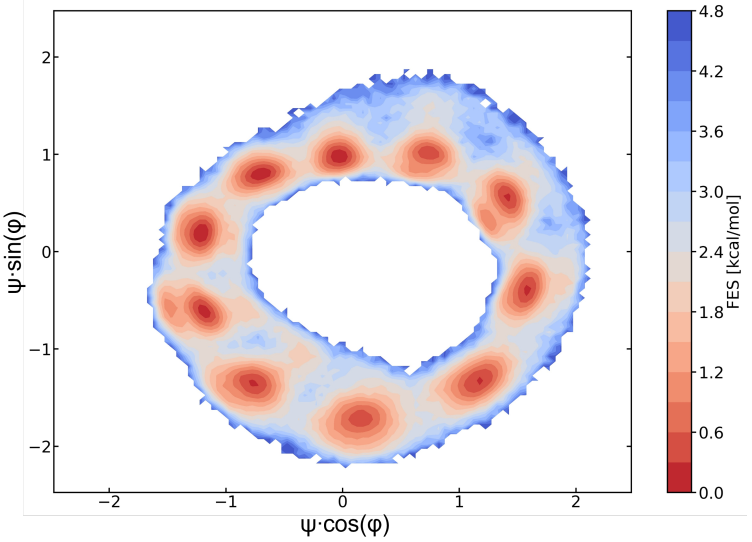

Figure S10: Free Energy Surface of CPP angular configuration as a function of ϕ and ψ angles.

Shuttling and kinetics of rigid bistable [2]Rotaxane

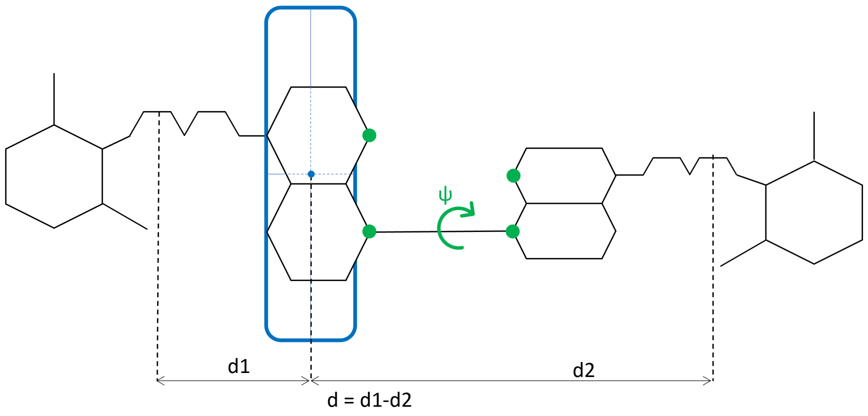

Figure S11: Schematic representation of WT-MetaD simulation setup for system **4**. The two selected CVs d and ψ are displayed.

The WT-MetaD simulation of system **4** has been run using the difference between the distance between the center of geometry of $CBPQT^{4+}$ and the oxygen atom of the last PEG of one lateral chain and the distance between the center of geometry of the ring and the oxygen atom of the last PEG of the other lateral chain (CV1) and the torsional angle ψ between the two naphthalenes (CV2) as CVs. A schematic representation of the CVs used for the WT-MetaD simulation of **4** is showed in Fig. S11. The bias has been added every 2 ps, using Gaussians with an initial height of 1 kJ/mol, a width of 0.2 nm for CV1 and 0.2 rad for CV2, and a bias factor of 20.

For estimating the kinetic rates, forty-five infrequent WT-MetaD have been run, using the distance between the center of geometry of $CBPQT^{4+}$ and the center of geometry of the closest naphthalene (CV1) and the torsional angle between the two naphthalenes (CV2) as collective variables. The bias has been added every 20 ps, using Gaussians with an initial height of 0.239 kJ/mol, a width of 0.1 nm for CV1 and 0.2 rad for CV2, and a bias factor of 20. The simulations ended when $CBPQT^{4+}$ jumped from station 1 to station 2. The obtained ECDF and the fitting with the Poissonian distribution for **4** is showed in Fig. S12.

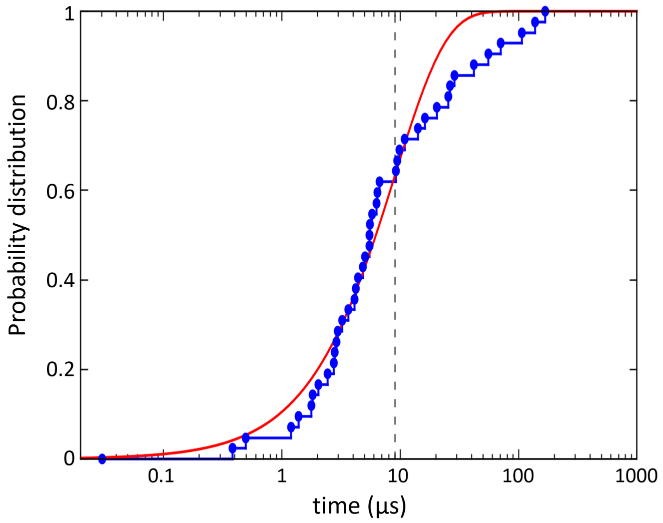

Figure S12: Transition times estimated via multiple infrequent metadynamics simulations (blue steps) and Poissonian probability distribution (red curve) for the transition of $CBPQT^{4+}$ from one naphthalene to the other in system **4**. The vertical dashed line represents the characteristic timescale τ associated with the event and is estimated by fitting the ECDF with the Poissonian distribution.

Table S1: Details of Molecular Systems simulated in this work

**System**	**Solvent**	**Number of atoms**	**Size of the box (Å)**	**Barostat**	**Simulation time (μs)**
**1**	ACN	26162	8.2x7.4x6.7	Parr-Rahm	2
**1**	$CHCl_3$	26621	10.6x9.1x7.6	Parr-Rahm	2
**1**	DCM	22676	9.5x8.1x6.8	Berendsen	2
**1**	DMSO	21732	7.5x6.4x5.3	Parr-Rahm	1.6
**2**	DCM	5495	5.6x4.8x4.5	Berendsen	1
**3**	$CHCl_3$	21849	9.7x7.8x7.8	Berendsen	2
**4**	ACE	11724	8.9x6.9x6.9	Parr-Rahm	2

Mapping auxiliary variables in CV space with conditional probability

In this paper, we used the approach described by Gimondi et Al.[22] to build maps of auxiliary variables in CV space (e.g. number of hydrogen bonds, SASA, enthalpy and entropy).

Adopting the notation of the reference paper, $\mathbf{s}$ is the set of CVs where the system is mapped, and $\bar{s}$ is the auxiliary variable. Each point in $\mathbf{s}$ represents an ensemble of degenerate configurations. One can define a local probability density for $\bar{s}$, for any values of $\mathbf{s}$, namely the conditional probability density of $\bar{s}$ under a constraint on the value of $\mathbf{s}$:

$$p(\bar{s}|\mathbf{s}) = \frac{\displaystyle\int e^{-\beta F(\mathbf{s}|\bar{s})}\delta(\mathbf{s}-\mathbf{s'})\delta\mathbf{s'}}{\displaystyle\iint e^{-\beta F(\mathbf{s}|\bar{s})}\delta(\mathbf{s}-\mathbf{s'})d\bar{s}d\mathbf{s}} \tag{S5}$$

From the probability density, $p(\bar{s}|\mathbf{s})$ one can compute the ensemble average of $\bar{s}$ over the ensemble of configurations degenerates in $\mathbf{s}$:

$$\langle\bar{s}\rangle_{\mathbf{s}} = \int \bar{s}p(\bar{s}|\mathbf{s})d\bar{s} \tag{S6}$$

Enthalphic and entropic contribution to FES

The WT-MetaD simulations allow us to compute the projection of the free-energy surface on the space of the collective variables. The Gibbs free-energy can be written as the sum of the enthalpic and the entropic part:

$$\Delta G(\mathbf{s}) = \Delta H(\mathbf{s}) - T\Delta S(\mathbf{s}) \tag{S7}$$

The approach proposed in Ref. 22 and recently applied for studying the early stage of nucleation of metal organic frameworks[24] was employed here for a systematic breakdown of the free energy surface. The enthalpic contribution to the free energy is:

$$\Delta H(\mathbf{s}) = \Delta U(\mathbf{s}) + P\Delta V(\mathbf{s}) \tag{S8}$$

where P is the pressure and V($\mathbf{s}$) is the volume, and U($\mathbf{s}$) is the sum of the ensemble averages

of the potential and kinetic energy:

$$U(\mathbf{s}) = \langle E^P \rangle_{\mathbf{s}} + \langle E^K \rangle_{\mathbf{s}} \tag{S9}$$

At this point, it is worth noting that for negligible variations of the volume mapped on the CV space, the term PΔV($\mathbf{s}$) is constant, and the enthalpy reduces to the ensemble average of the internal energy on $\mathbf{s}$ U($\mathbf{s}$). Moreover, at constant temperature, the kinetic energy does not depend on $\mathbf{s}$, and the internal energy reduces to the potential energy of the system $\Delta E_{P(\mathbf{s})}$.

For systems immersed in explicit solvent the potential energy of the system can be further decomposed as follows:

$$\Delta E_P(\mathbf{s}) = \Delta\langle E_P^{Solute} \rangle_{\mathbf{s}} + \Delta\langle E_P^{Solvent} \rangle_{\mathbf{s}} + \Delta\langle E_P^{Solute-Solvent} \rangle_{\mathbf{s}} \tag{S10}$$

The equation S10 can be reduced, noting that the term $\Delta\langle E_P^{Solvent} \rangle_{\mathbf{s}}$ is independent from the value of the CV, $\mathbf{s}$.

In conclusion, the enthalpy mapped on the CV space reduces to the ensemble average of the potential energy of the system along the CV, and the entropy can be obtained by difference.

Calculation of the rotation in [10]CPP-Fullerene [2]Rotaxane

The analysis of the dynamics of the [10]CPP over the central Fullerene was done by decomposing its rotation (ϕ – rotation axis of the [10]CPP parallel to cylindrical symmetry axis of the molecule itself) from the tilting (ψ – tilting axis orthogonal to the cylindrical symmetry axis of [10]CPP) (see Fig. 4d in main text). Performing principal component analysis (PCA) over $\cos(\phi)$, $\sin(\phi)$, $\cos(\psi)$ and $\sin(\psi)$ sampled during unbiased MD, the first two principal components, PC1 and PC2 (the new coordinates obtained from the analysis), transform the displacement of the measured values into ten blobs over a circle (see Fig. 4e in main text the $-K_BT \log(\rho)$ map, where ρ is the density map of the configurations). The position along

the circle is directly related to ϕ as shown in Fig. 4e, while the radial position correlates with ψ. By performing cluster analysis by the means of Probabilistic Analysis of Molecular Motifs (PAMM),[25] it clearly identifies ten ensembles, and by analysing the dynamics time evolution of the labelling of each data-point, we can measure the ~ 0.1ns residence time in each cluster (configurations), and then moving to one of the next neighbour clusters. From this analysis it is clear that the most relevant movement of [10]CPP is the rotation, while the tilting mainly fluctuates about its equilibrium position.

References

(1) Wang, J.; Wolf, R. M.; Caldwell, J. W.; Kollman, P. A.; Case, D. A. Development and testing of a general amber force field. *J. Comput. Chem.* **2004**, *25*, 1157–1174.

(2) Bayly, C. I.; Cieplak, P.; Cornell, W.; Kollman, P. A. A well-behaved electrostatic potential based method using charge restraints for deriving atomic charges: the RESP model. *J. Phys. Chem.* **1993**, *97*, 10269–10280.

(3) Frisch, M. J. et al. Gaussian˜16 Revision C.01. 2016; Gaussian Inc. Wallingford CT.

(4) Jakalian, A.; Bush, B. L.; Jack, D. B.; Bayly, C. I. Fast, efficient generation of high-quality atomic charges. AM1-BCC model: I. Method. *Journal of Computational Chemistry* **2000**, *21*, 132–146.

(5) Jakalian, A.; Jack, D. B.; Bayly, C. I. Fast, efficient generation of high-quality atomic charges. AM1-BCC model: II. Parameterization and validation. *Journal of Computational Chemistry* **2002**, *23*, 1623–1641.

(6) Case, D. A.; Aktulga, H. M.; Belfon, K.; Ben-Shalom, I.; Brozell, S. R.; Cerutti, D. S.; Cheatham III, T. E.; Cruzeiro, V. W. D.; Darden, T. A.; Duke, R. E.; others *Amber 2021*; University of California, San Francisco, 2021.

(7) Caleman, C.; van Maaren, P. J.; Hong, M.; Hub, J. S.; Costa, L. T.; van der Spoel, D. Force Field Benchmark of Organic Liquids: Density, Enthalpy of Vaporization, Heat Capacities, Surface Tension, Isothermal Compressibility, Volumetric Expansion Coefficient, and Dielectric Constant. *J. Chem. Theory Comput.* **2012**, *8*, 61–74.

(8) van der Spoel, D.; van Maaren, P. J.; Caleman, C. GROMACS molecule & liquid database. *Bioinformatics* **2012**, *28*, 752–753.

(9) Bussi, G.; Donadio, D.; Parrinello, M. Canonical sampling through velocity rescaling. *J. Chem. Phys.* **2007**, *126*, 014101.

(10) Berendsen, H.; Postma, J.; van Gunsteren, W.; Di Nola, A.; Haak, J. Molecular dynamics with coupling to an external bath. *J. Chem. Phys.* **1984**, *81*, 3684–3690.

(11) Parrinello, M.; Rahman, A. Polymorphic transitions in single crystals: A new molecular dynamics method. *J. Appl. Phys.* **1981**, *52*, 7182–7190.

(12) Darden, T.; York, D.; Pedersen, L. Particle mesh Ewald: An N·log(N) method for Ewald sums in large systems. *J. Chem. Phys.* **1993**, *98*, 10089–10092.

(13) Hess, B.; Bekker, H.; Berendsen, H. J. C.; Fraaije, J. G. E. M. LINCS: A linear constraint solver for molecular simulations. *J. Comput. Chem.* **1997**, *18*, 1463–1472.

(14) Abraham, M. J.; Murtola, T.; Schulz, R.; Páll, S.; Smith, J. C.; Hess, B.; Lindahl, E. GROMACS: High performance molecular simulations through multi-level parallelism from laptops to supercomputers. *SoftwareX* **2015**, *1-2*, 19–25.

(15) Promoting transparency and reproducibility in enhanced molecular simulations. *Nat. Methods* **2019**, *16*, 670–673.

(16) Tribello, G. A.; Bonomi, M.; Branduardi, D.; Camilloni, C.; Bussi, G. PLUMED 2: New feathers for an old bird. *Comput. Phys. Commun.* **2014**, *185*, 604–613.

(17) Tiwary, P.; Parrinello, M. From Metadynamics to Dynamics. *Phys. Rev. Lett.* **2013**, *111*, 230602.

(18) Salvalaglio, M.; Tiwary, P.; Parrinello, M. Assessing the Reliability of the Dynamics Reconstructed from Metadynamics. *J. Chem. Theory Comput.* **2014**, *10*, 1420–1425.

(19) Resnick, S. *Adventures in Stochastic Processes*; Adventures in Stochastic Processes; Birkhäuser Boston, 1992.

(20) Jr., F. J. M. The Kolmogorov-Smirnov Test for Goodness of Fit. *J. Am. Stat. Assoc.* **1951**, *46*, 68–78.

(21) Miller, L. H. Table of Percentage Points of Kolmogorov Statistics. *J. Am. Stat. Assoc.* **1956**, *51*, 111–121.

(22) Gimondi, I.; Tribello, G. A.; Salvalaglio, M. Building maps in collective variable space. *J. Chem. Phys.* **2018**, *149*, 104104.

(23) Xu, Y.; Kaur, R.; Wang, B.; Minameyer, M. B.; Gsänger, S.; Meyer, B.; Drewello, T.; Guldi, D. M.; von Delius, M. Concave–convex Π–Π template approach enables the synthesis of [10] cycloparaphenylene–fullerene [2] rotaxanes. *J. Am. Chem. Soc.* **2018**, *140*, 13413–13420.

(24) Kollias, L.; Cantu, D. C.; Glezakou, V.-A.; Rousseau, R.; Salvalaglio, M. On the Role of Enthalpic and Entropic Contributions to the Conformational Free Energy Landscape of MIL-101(Cr) Secondary Building Units. *Adv. Theory Simul.* **2020**, *3*, 2000092.

(25) Gasparotto, P.; Meißner, R. H.; Ceriotti, M. Recognizing local and global structural motifs at the atomic scale. *J. Chem. Theory Comput.* **2018**, *14*, 486–498.
